# Identifying areas of degrading and improving groundwater-quality conditions in the State of California, USA, 1974–2014

**DOI:** 10.1007/s10661-020-8180-y

**Published:** 2020-03-25

**Authors:** Bryant C. Jurgens, Miranda S. Fram, Jeffrey Rutledge, George L. Bennett V.

**Affiliations:** US Geological Survey, 6000 J St Placer Hall, Sacramento, CA 95819 USA

**Keywords:** Groundwater, Trends, Water quality, Spatial aggregation, Nitrate

## Abstract

Areas of improving and degrading groundwater-quality conditions in the State of California were assessed using spatial weighting of a new metric for scoring wells based on constituent concentrations and the direction and magnitude of a trend slope (Sen). Individual well scores were aggregated across 2135 equal-area grid cells covering the entire groundwater resource used for public supply in the state. Spatial weighting allows results to be aggregated locally (well or grid cell), regionally (groundwater basin), provincially, or statewide. Results differentiate degrading (increasing concentration trends) areas with low to moderate concentrations (unimpaired) from degrading areas with moderate to high concentrations (impaired). Results also differentiate improving areas (decreasing concentration trends) in the same manner. Multi-year to decadal groundwater-quality trends were computed from periodic, inorganic water-quality data for 38 constituents collected between 1974 and 2014 for compliance monitoring of nearly 13,000 public-supply wells (PSWs) in the State of California. Mann-Kendall (MK) rank correlations and Sen’s slope estimator were used to detect statistically significant trends for the entire period of recorded data (long-term trend), for the period since 2000 (recent trend), for different pumping seasons (seasonal trend), and for reversals of trends. Statewide, the most frequently detected trends since 2000 were for nitrate (36%), gross alpha/uranium (10%), arsenic (14%), total dissolved solids (TDS) (23%), and the major ions that contribute to TDS (19–28%). The Transverse and Selected Peninsular Ranges (TSPR) and the San Joaquin Valley (SJV) hydrogeologic provinces had the largest percentage of areas with moderate to high nitrate concentrations and groundwater quality trends. Improving nitrate concentrations in parts of the TSPR is associated with long-term managed aquifer recharge that has replaced historical, agriculturally affected groundwater with low-nitrate recharge in parts of the TSPR. This example suggests that application of dilute, excess surface water to agricultural fields during the winter could improve groundwater-quality in the SJV over the long term.

**Electronic supplementary material:**

The online version of this article (10.1007/s10661-020-8180-y) contains supplementary material, which is available to authorized users.

## Introduction

Groundwater-quality trends are often evaluated on a well-by-well basis where statistical tests or linear regression is applied to water-quality monitoring data to detect a trend and compute a rate of change. The results provide an overall indication of whether water quality at a well is improving, degrading, or is static. For government entities tasked with assessing groundwater-quality degradation or improvement and with evaluating the effectiveness of management solutions on regional to statewide scales, there is a need to aggregate well-specific trends and concentrations at larger spatial scales so that unbiased, inter- and intra-basin comparisons can be made to help guide priorities and management decisions.

Regional factors such as changes in land use and sources of recharge often influence groundwater-quality trends at wells in addition to localized factors such as well construction characteristics and pumping. For example, regional nitrate trends have been found in many aquifers throughout the world and these trends have been linked to changes in land use patterns and nitrate inputs (Broers and van der Grift 2004; Stuart et al. 2007; Visser et al. 2007; Hansen et al. 2011; Kent and Landon 2013; Burow et al. 2013; Lopez et al. 2015). Land use practices can also alter the natural chemistry of water that recharges an aquifer and cause trace elements that are naturally present, like uranium, to become mobilized (Jurgens et al. 2010; Ayotte et al. 2011). Short-term, cyclical pumping patterns resulting from semi-annual water demand can also lead to seasonal water-quality variations in wells (Bexfield and Jurgens 2014). On longer time scales, groundwater-quality trends may be caused by regional pumping patterns that alter the origin of groundwater reaching wells (Starn et al. 2014). In many aquifers where contaminant loading has affected groundwater quality, different well construction characteristics and positions within the flow system (horizontally or vertically) can yield contrasting water-quality trends (Böhlke 2002; Broers and van der Grift 2004; Kent and Landon 2013; Böhlke et al. 2014).

Although the aggregation of well-specific trend results has been done to characterize regional tendencies of nitrate for hydrogeologic regions or basins (Stuart et al. 2007; Lopez et al. 2015) and US counties (Helsel and Frans 2006), there also is a need to consider the concentration in conjunction with the rate of change in order to prioritize areas that have high concentrations and are degrading rapidly over areas that have low concentrations and are degrading at a slower rate. Aggregation of concentration and rate of change into a single metric that can be applied at multiple scales has not been done.

**Fig. 1:**
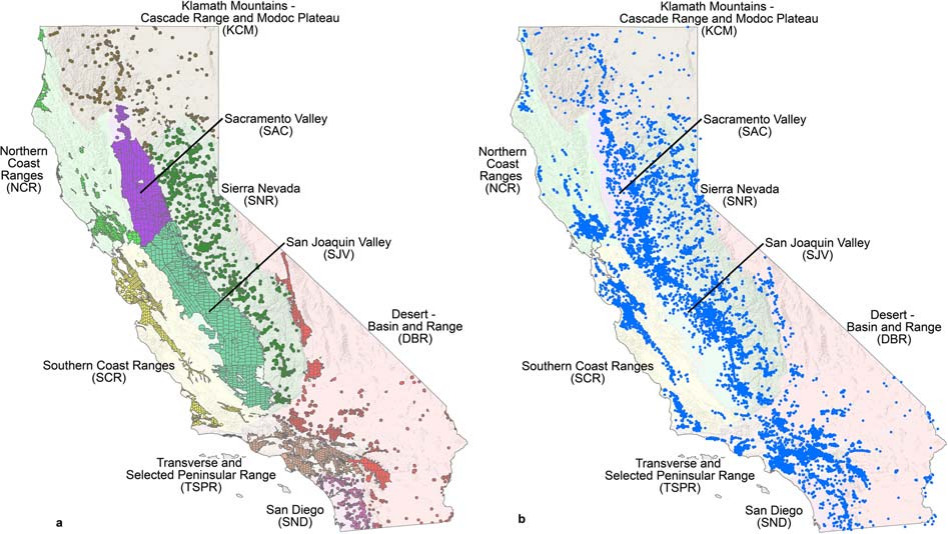
Map of California showing boundaries of cells (**a**) and wells (**b**) located within nine hydrogeologic provinces of the state assessed in this study

In California, recent groundwater legislation (Sustainable Groundwater Management Act) has mandated the formation of local groundwater sustainability agencies to assess, plan, monitor, and implement changes to sustainably manage California’s groundwater basins, including prevention of groundwater quality degradation (California Department of Water Resources 2015). In 2014, the State of California had over 15,000 active, inactive, and standby public-supply wells (California State Water Resources Control Board – Division of Drinking Water (SWRCB-DDW) 2016) (Fig. 1) that provided 45% of the public water supply for 38 million people (Dieter et al. 2018). Beginning in the mid-1970s, the U.S. Environmental Protection Agency (USEPA) and U.S. State agencies have required periodic testing of public drinking water sources for a wide range of regulated and unregulated water-quality constituents. Although the monitoring data are intended for regulatory compliance with water-quality benchmarks, they also record changes in the quality of the water resource over time. Consequently, these data can be used by local groundwater sustainability agencies and the State of California to assess groundwater-quality degradation or improvement in groundwater basins throughout California. To accomplish these goals and compare results across California, robust and consistent techniques for processing, analyzing, and detecting water-quality trends and methods to organize the results are needed.

The most widely used statistical test for detecting trends is the Mann-Kendall (MK) test for monotonic trends (Kendall 1938, 1975; Mann 1945). This test has been adapted to assess trends in data with underlying seasonal patterns (Hirsch et al. 1982) and to assess whether multiple sites located in the same region or area have a consistent trend direction (regional MK test, Helsel and Frans 2006). Though the computation of the MK test is straightforward, the water-quality data collected from monitoring programs often require screening because of temporal changes in analytical reporting levels (Hirsch et al. 1982; Hirsch and Slack 1984) or require regularization because of serial dependence caused by varying sample frequency (Hirsch and Slack 1984; Hirsch et al. 1991; Wahlin and Grimvall 2010). In addition, the presence of equal values or “ties” in water-quality data that occurs from reporting levels, screening levels, or rounding of analytical results can make the detection of statistical significance more difficult (Amerise and Tarsitano 2016). Inconsistent or inappropriate methods used to deal with any one of these issues by local agencies or water purveyors when computing trends can hinder the assessment of regional and statewide trends.

The California Groundwater Ambient Monitoring and Assessment Program Priority Basin Project (GAMA-PBP) recently completed a statewide assessment of the status of water quality in groundwater resources used for public drinking water (Belitz et al. 2015). The GAMA-PBP is part of the California State Water Resources Control Board (SWRCB) GAMA program (SWRCB 2018). The GAMA-PBP assessment found that 18.9% of the area of groundwater resources used for public drinking water had trace elements present at concentrations greater than a health-based benchmark (USEPA or SWRCB-DDW maximum contaminant level (MCL) or action level (AL), or USEPA lifetime health advisory level (HAL) (SWRCB-DDW 2018; USEPA 2018a, b), and 4.1% of the area had nitrate concentrations greater than the MCL.

In this paper, the assessment of Belitz et al. (2015) is extended to incorporate information about trends in groundwater quality in aquifers used for public drinking water supply. The purpose of this paper is to (1) provide a new method for scoring wells based on constituent concentrations and trend direction and magnitude and (2) using this method, to examine and interpret score patterns across several spatial scales in California.

## Methods

Areas of degrading or improving groundwater quality conditions were assessed at different spatial scales within the State of California using a network of 2135 equal-area grid cells , covering an area of about 105,312 km^2^. The grid cell network is the same used by Belitz et al. (2015) and is available digitally from Johnson et al. (2018) (Fig. 1). The grid cells encompass nine hydrogeologic provinces in California: Desert – Basin and Range (DBR), Klamath Mountains - Cascade Range and Modoc Plateau (KCM), Northern Coast Ranges (NCR), Sacramento Valley (SAC) , San Diego (SND), San Joaquin Valley (SJV), Sierra Nevada (SNR), Southern Coast Ranges (SCR), and Transverse and Selected Peninsular Ranges (TSPR). The provines are composed of 87 study areas that correspond to California Department of Water Resources groundwater basins (California Department of Water Resources 2003) or areas outside of groundwater basins. The study areas investigated by Belitz et al. (2015) included 95% of the area statewide where public-supply wells (PSWs) are located and 99% of the population supplied by PSWs. Therefore, the gridded area in Fig. 1 is essentially the entire area of the groundwater resource used for public supply in California.

The following sections describe a semi-automated routine that was developed to (1) process water-quality time series records to reduce serial dependence and normalize the data for changing reporting levels, (2) compute the MK test for different trends and check for statistical significance, (3) compute well and cell scores, and (4) compute aggregated results for study areas, hydrogeologic provinces, and the state.

### Data compilation

Groundwater-quality data for 38 inorganic constituents were analyzed for trends (Table 1). Data were compiled from the California State Water Resources Control Board Division of Drinking Water (SWRCB-DDW) database of water quality collected for compliance purposes from 1974 thru 2014 (SWRCB-DDW 2016) and from data collected by the U.S. Geological Survey (USGS) GAMA-PBP from 2004 thru 2014 (Jurgens et al. 2018). More than 95% of the data used for trends were from the SWRCB-DDW database. Data from the USGS GAMA program supplements the SWRCB-DDW data, particularly in rural areas of California where water-quality monitoring is not as frequent. The SWRCB-DDW data are available from the SWRCB’s GeoTracker GAMA on-line groundwater information system (California State Water Resources Control Board 2018); data from the USGS are available on-line from USGS National Water Information System (NWIS) database (USGS 2018) and the USGS GAMA-PBP web mapper (Jurgens et al. 2018).

**Table 1:** List of water-quality constituents analyzed for trends with the number of wells with at least one sample, the constituent screening level, and water-quality benchmark

Constituent	Number of wells in gridded area with at least one sample	SWRCB-DDW STORET parameter code	GAMA-PBP USGS parameter code	Units	Most frequent SWRCB-DDW reporting limit	Benchmark type^e^	Benchmark value
Nutrients							
Nitrate	15,476	71850^a^	00618, 00631	mg/L as N	0.452	MCL-US	10
Nitrite	13,646	00620^b^	00613	mg/L as N	0.4	MCL-US	1
Radioactive constituents							
Gross alpha	12,100	01501	62636	pCi/L	3	MCL-US	15
Gross beta	2913	03501	62642	pCi/L	1	MCL-CA	50
Radium 226	3930	09501	09511	pCi/L	1	MCL-US	5
Radium 228	8221	11501	81366	pCi/L	1	MCL-US	5
Radium 226 + 228	1580	11503	09511 + 81366	pCi/L	1	MCL-US	5
Uranium	7117	28012	22703^c^	pCi/L	2	MCL-CA	20
Trace elements							
Aluminum	12,479	01105	01106	μg/L	50	MCL-CA	1000
Antimony	11,776	01097	01095	μg/L	6	MCL-US	6
Arsenic	12,998	01002	01000	μg/L	2	MCL-US	10
Barium	12,853	01007	01005	μg/L	100	MCL-CA	1000
Beryllium	11,703	01012	01010	μg/L	1	MCL-US	4
Boron	8844	01020	01020	μg/L	100	HAL-CA	6000
Cadmium	12,859	01027	01025	μg/L	1	MCL-US	5
Chromium (total)	12,854	01034	01030	μg/L	10	MCL-CA	50
Copper	12,642	01042	01040	μg/L	50	AL-US	1300
Fluoride	13,465	00951	00950	mg/L	0.1	MCL-CA	2
Iron	13,298	01045	01046	μg/L	50	SMCL-CA	300
Lead	12,591	01051	01049	μg/L	5	AL-US	15
Manganese	13,296	01055	01056	μg/L	30	SMCL-CA	50
Mercury	12,733	71900	71890	μg/L	1	MCL-US	2
Nickel	11,798	01067	01065	μg/L	10	MCL-CA	100
Selenium	12,855	01147	01145	μg/L	5	MCL-US	50
Silver	12,722	01077	01075	μg/L	10	SMCL-CA	100
Thallium	11,713	01059	01057	μg/L	1	MCL-US	2
Vanadium	8212	01087	01085	μg/L	50	NL-CA	500
Zinc	12,698	01092	01090	μg/L	50	HAL-CA	2000
Major ions, pH, TDS, and hardness							
Alkalinity	13,012	00410	39086	mg/L as CaCO_3_	5	None	None
Calcium	13,148	00916	00915	mg/L	5	None	None
Chloride	12,716	00940	00940	mg/L	1	SMCL-CA	500^d^
Magnesium	13,133	00927	00925	mg/L	2	None	None
Potassium	11,629	00937	00935	mg/L	5	None	None
Sodium	13,125	00929	00930	mg/L	0.5	None	None
Sulfate	12,744	00945	00945	mg/L	2	SMCL-CA	500^d^
pH, Lab	13,176	00403	00400	Unitless	0	SMCL-US	6.5–8.5
Total dissolved solids (TDS)	12,611	70300	70300	mg/L	3	SMCL-CA	1000^d^
Hardness	13,134	00900	00900	mg/L as CaCO_3_	20	None	None

^a^Nitrate is reported in the SWRCB-DDW database as parameter code 71850 in units of mg/L as nitrate (NO3). The data are converted to units of mg/L as nitrogen (N) for this study

^b^Nitrite is reported in the SWRCB-DDW database as parameter code 00620 in units of μg/L as nitrogen. The data are converted to units of mg/L as nitrogen for this study

^C^Uranium is reported in the USGS NWIS database (USGS 2018) as parameter code 22703 in units of μg/L. The data are converted to units of pCi/L using a conversion factor of 0.79 pCi/μg for this study

^d^Chloride, sulfate, and TDS have recommended lower and upper SMCL benchmarks. The upper benchmarks are used for this study

^e^Benchmarks were selected in the following order of priority: (1) U.S. Environmental Protection Agency (USEPA) or California State Water Resources Control Board Division of Drinking Water (SWRCB-DDW) maximum contaminant levels (MCL) or action levels (AL), whichever has the lowest concentration (USEPA 2018a, b; SWRCB-DDW, 2018); (2) SWRCB-DDW secondary maximum contaminant levels (SMCL) [SWRCB-DDW 2018]; (3) USEPA lifetime health advisory levels (HAL); (4) SWRCB-DDW notification level response level (NL-RL)

The data used for trends are from sample points that discharge raw, untreated groundwater. This analysis does not evaluate trends in water delivered to consumers, which may be treated or blended with other water before delivery to consumers. The data collected by water purveyors and reported to the state was not evaluated for contamination, bias, or analytical quality. Data reported to the State of California are from unfiltered samples and values for pH are laboratory values, so data from the SWRCB-DDW database (SWRCB-DDW 2016) may not fully represent ambient groundwater-quality conditions. USGS samples were collected in accordance with protocols established by the USGS National Field Manual (USGS 2018) and the USGS National Water Quality Assessment (NAWQA) project (Koterba et al. 1995). USGS sampling protocols are designed to obtain samples that represent conditions in the aquifer.

In California, PSWs are wells belonging to systems that serve 25 or more people or have 15 or more service connections (SWRCB-DDW 2016). Most PSWs in the SWRCB-DDW database are community wells (cities, towns, and mobile-home parks) but also include non-transient, non-community wells (schools, workplaces, and restaurants) and transient, non-community wells (campgrounds, parks, and highway rest areas).

The number of PSWs with water-quality data reported in the SWRCB-DDW database increased from about 100 wells per year in the early 1980s to about 9000 wells per year in 2002–2014 (Fig. S1). PSWs classified as non-community and community wells belonging to smaller systems generally have fewer samples than community wells from larger systems, especially for constituents other than nitrate. Consequently, small-system wells are less likely to have sufficient number of data points in the SWRCB-DDW database for trend analysis. To reduce this potential bias, data from 1544 PSWs sampled for the GAMA-PBP assessment between 2004 and 2015 were included. Most of these sites were sampled once, and about 400 were sampled at least twice during that period. Because the GAMA-PBP sampled both community and non-community PSWs, combining the GAMA-PBP and SWRCB-DDW datasets increased the number of PSWs with sufficient number of data points for trends analysis.

### Statistical methods

The MK rank correlation (Kendall 1938, 1975; Mann 1945), which is a non-parametric, rank-based statistical test, and Sen’s slope estimator (Sen 1968) were used to assess trends in water-quality data. Trends were accepted as statistically significant when MK rank correlation *p* values were below a significance level (*α*) of 0.1 and the Sen’s slope estimator was not zero. Positive Sen’s slopes indicate increasing concentrations while negative slopes indicate decreasing concentrations. Tests were computed using the Python scripting language (PSF 2019) for constituents at wells with four or more laboratory analyses that spanned at least 5 years. Four unique values (no ties present) is the minimum number of data points necessary to achieve a *p* value (0.0833) less than the significance level.

Before a statistical test was applied, water-quality data were processed to reduce biases in trend detection caused by serial correlation, changing reporting levels, and seasonal patterns (see SI). In general, the most common detection level reported with the SWRCB-DDW data was used as a truncation level such that non-detections and concentrations below the truncation level were recoded to the most common detection level for each constituent listed in Table 1. Non-detect values above the truncation level were removed from the dataset. To reduce the effects of serial correlation and to test for trends in data that display significant water-quality differences among pumping seasons, water-quality data were classified as a *Summer* sample if the sample date was between May 1st and October 31st or a *Winter* sample if the sample date was outside the *Summer* date range. For each season, the median concentration and median date for summer and winter samples were used when more than one result was measured in a season. This method produces at most two data points for each year (see Fig. S3 in supplemental material).

**Fig. 2:**
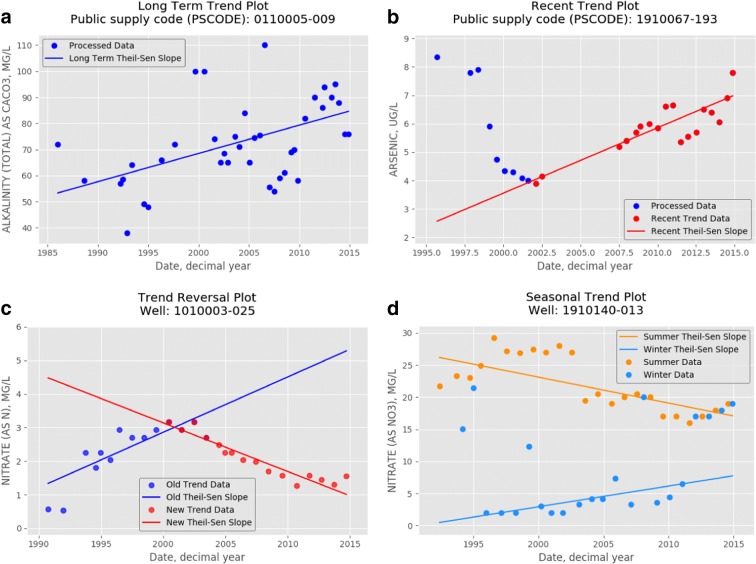
Examples of **a** long-term, **b** recent, **c** reversal, and **d** seasonal trends

Tests for trends were applied to different time periods to identify long-term trends (LTTs), recent trends (RTs), reversals in trends (TRVs), and trends that have seasonal concentration differences (Fig. 2). The entire period of recorded data was used to identify LTTs. whereas RTs were evaluated with water quality data collected since the year 2000. For RTs, the set of most recent data points with the steepest Sen’s slope since year 2000 is recorded and plotted with red circles (Fig. 2). LTTs and RTs were computed for datasets with four or more unique processed analyses and each set of data was required to span at least 5 years. LTTs include data from wells and areas that may no longer be used and therefore provide a more complete picture of concentration trends over the entire history of data reported to the state, whereas RTs reflect trends of the groundwater resources currently being used over the last 15-year period. Because groundwater moves slowly and because many inorganic constituents are not required for sampling on an annual basis, the 15-year window provides enough time and data to be collected to allow trends testing.

The TRVs show a change in trend direction either from decreasing to increasing or from increasing to decreasing concentration trend. TRVs may be useful for identifying areas where changes in land use, hydrology (e.g., recharge rates and sources of recharge), and source loading or contaminant regulation have led to substantial changes in concentrations in an aquifer. TRVs were computed for datasets with at least 8 data points spanning at least 10 years. TRVs were determined by looking for opposite trends in two continuous segments; one segment from the oldest data and one segment from the newest data. To determine if a change in slope occurred, the MK test was computed multiple times by incrementally varying the size of the oldest (*S*_old_ = *i*) and newest (*S*_old_ = *N* − *i*) segments, when *i* goes from 4 to the number of data points (*N*). Because this analysis can produce multiple sets of segments with TRVs around the inflection point, the set of newest data with the largest change in trend slope was reported (Fig. 2). This procedure identifies trends that have reversed direction once over the entire period of record rather than trends with frequent reversals caused by variability over shorter durations (< 5 years).

Seasonal trends can result from natural seasonal recharge cycles (Stuart et al. 2007) or from cyclical periods of pumping and non-pumping that cause changes in the water sampled by a well (Bexfield and Jurgens 2014). Trends can be masked, and the rate of change can be over/underestimated, by seasonal differences in water-quality data (Hirsch et al. 1982; Helsel and Hirsch 1995). Seasonality was identified using the Mann-Whitney test (Mann and Whitney 1947) for differences between seasonal populations of water-quality data when there were at least four analyses in each season. If differences in concentrations between seasons were significant, MK rank correlation and Sen’s slope estimator were computed for each set of seasonal data. A seasonal trend was statistically significant if at least one MK test *p* value was below the significance level and the Sen’s Slope estimator was not zero. This approach to seasonal trends is different than the computation by the Seasonal MK trend test (Hirsch et al. 1982), which is a sum of the individual Kendall’s *S* statistic among seasons and generally requires trends to be in the same direction for most seasons to be significant.

### Water-quality benchmarks

Constituent concentrations were compared to federal and state water-quality benchmarks (Table 1). Benchmarks were selected in the following order of priority: The USEPA or SWRCB-DDW maximum contaminant level (MCL) or action level (AL), whichever had the lowest concentration (24 constituents), SWRCB-DDW secondary maximum contaminant levels (SMCL; the upper SMCL was used for constituents with lower and upper recommended values; 5 constituents), HAL (2 constituents), then SWRCB-DDW notification level–response level, NL-RL (1 constituent) (USEPA 2018a, b; SWRCB-DDW 2018). Sample concentrations (*C*) are defined as “high,” “moderate,” and “low” relative to the benchmark concentration (*B*): High C > B; Moderate B/2 < C ≤ B; Low C ≤ B/2.

Six constituents did not have a benchmark, but these constituents may contribute or explain trends of other constituents with benchmarks. For example, calcium does not have a benchmark but contributes to total dissolved solid (TDS) concentrations so trends in calcium concentrations may partly explain TDS trends. Thus, they are evaluated for trends but not for the combined metric of improving and degrading groundwater-quality conditions.

### Well and cell scores

Each well was scored (*S*_well_) for the concentration (*C*) relative to its benchmark (*B*) and scored for the magnitude and direction of the Sen Slope (*SS*) trend relative to half the benchmark. Well scores were computed for constituents with water-quality benchmarks using the most recent measured concentration. The time required for the concentration to increase by a magnitude of half the benchmark (*T*_hb_), the concentration score (*S*_*C*_), and the trend score (*S*_*T*_) is calculated as

1 $$ {\mathrm{T}}_{\mathrm{hb}}=\frac{0.5B}{SS} $$

2 $$ {S}_C=\left\{\begin{array}{c}0.5,\kern0.5em if\ \frac{C}{B}<0.5\\ {}\kern3.5em 1,\kern0.75em if\ 0.5\le \frac{C}{B}<1\\ {}\begin{array}{cc}1.5,& if\ \frac{C}{B}\ge 1\end{array}\end{array}\right. $$

3 $$ {S}_T=\left\{\begin{array}{c}0,\kern0.5em \mathrm{if}\ \mathrm{no}\ \mathrm{trend}\\ {}\operatorname{sign}(SS)\ast 0.5,\kern0.5em \mathrm{if}\ {T}_{\mathrm{hb}}<5\ \mathrm{years}\\ {}\operatorname{sign}(SS)\ast 0.4,\kern0.5em \mathrm{if}\ {T}_{\mathrm{hb}}<10\ \mathrm{years}\\ {}\operatorname{sign}(SS)\ast 0.3,\kern0.5em \mathrm{if}\ {T}_{\mathrm{hb}}<25\ \mathrm{years}\\ {}\operatorname{sign}(SS)\ast 0.2,\kern0.5em \mathrm{if}\ {T}_{\mathrm{hb}}<50\ \mathrm{years}\\ {}\operatorname{sign}(SS)\ast 0.1,\kern0.5em \mathrm{if}\ {T}_{\mathrm{hb}}\ge 50\ \mathrm{years}\end{array}\right. $$

4 $$ {S}_{\mathrm{well}}=\operatorname{sign}\left({S}_T\right)\ast \left({S}_C+{S}_T\right) $$

Wells with negative trends (decreasing concentrations) can have *S*_*T*_ ranging from 0 to − 1.5, wells with no trends (no significant changes in concentrations) can have values of 0.5, 1.0, or 1.5, and wells with positive trends (increasing concentrations) can have *S*_*T*_ ranging from 0.5 to 2.0. The well score is not symmetric, and all scores except − 1.0, − 0.5, 0.5, and 1.0 represent a unique combination of concentration and rate (Table 2).

**Table 2:** Matrix of possible well scores. Well scores move in opposite directions, such that wells with high concentrations and rapidly decreasing trends approach moderate scores while wells with high concentrations and rapidly increasing concentration trends approach a score of 2. Similarly, wells with low concentrations and improving conditions approach zero while wells with low concentrations and rapidly increasing concentration trends approach moderate scores

*T*_hb_, years	Concentration class	Hi	Mod	Low	Low	Mod	Hi
	*S*_*C*_	1.5	1	0.5	0.5	1	1.5
	Trend class	Improving			Degrading		
	Sign *S*_*T*_	−1	−1	-1	1	1	1
	|*S*_*T*_|	*S*_well_	*S*_well_	*S*_well_	*S*_well_	*S*_well_	*S*_well_
No trend	0	− 1.5	− 1	− 0.5	0.5	1	1.5
> 50	0.1	− 1.4	− 0.9	− 0.4	0.6	1.1	1.6
> 25	0.2	− 1.3	− 0.8	− 0.3	0.7	1.2	1.7
> 10	0.3	− 1.2	− 0.7	− 0.2	0.8	1.3	1.8
> 5	0.4	− 1.1	− 0.6	− 0.1	0.9	1.4	1.9
≤ 5	0.5	− 1	− 0.5	0	1	1.5	2

Similarly, a cell score, *S*_cell_, was calculated for each grid cell by using the concentration and trend score of each well in a cell, *n*:

5 $$ {S}_{\mathrm{cell}}=\frac{\operatorname{sign}\left({\sum}_{i=1}^n{S}_T\right)\ast \left({\sum}_{i=1}^n{S}_C+{\sum}_{i=1}^n{S}_T\right)}{n} $$

Cell scores that are negative indicate that water-quality trends are predominately improving whereas positive cell scores indicate that water-quality trends are predominately degrading. When positive and negative trend scores for wells in a cell are equal, the cell score is zero or indeterminate. Cell scores can be computed using any of the trend tests determined above; however, only recent trends results were used to compute cell scores because they provide the most recent picture of groundwater quality trends statewide.

Cell scores were classified into one of the nine categories: (1) not tested, (2) no trend, (3) improving (decreasing concentration trends) with high concentrations, (4) improving with moderate concentrations, (5) improving with low concentrations, (6) degrading (increasing concentration trends) with low concentrations, (7) degrading with moderate concentrations, (8) degrading with high concentrations, and (9) indeterminate.

### Aggregation

Spatial weighting was used to determine the areal proportion of the groundwater resource with trends and different classes of degradation or improvement in a study area or hydrogeologic province. Spatial weighting counteracts biases caused by differences in the spatial density of wells, so that areas with higher densities of wells or more frequent sampling will receive the same weight as other grid cells with lower densities of wells (Belitz et al. 2010).

Two types of aggregated results were determined. First, spatial weighting was used to determine the areal proportion of the groundwater resource in a study area, province, or state that had constituent concentration trends (*P*_*T*_). This is analogous to the spatial weighting used by Belitz et al. (2015) to calculate the areal proportion of the resource that has concentrations of a constituent in groundwater above a benchmark.

6 $$ {D}_T=\frac{N_T}{N_w} $$

7 $$ {P}_T=\frac{\sum_{i=1}^{n_c}{D}_{T,i}{A}_i}{\sum_{i=1}^{n_c}{A}_i} $$

where *N*_*w*_ is the number of wells in cell *i* that could be tested for trends (four or more data points), *N*_*T*_ is the number of wells in cell *i* with a trend (positive or negative), *D*_*T*_ is the detection frequency of a trend (positive or negative) among the wells in cell *i*, *A*_*i*_ is the area of cell *i*, and *n*_*c*_ is the number of cells in the study area, province, or state.

Second, spatial weighting was used to determine the areal proportion of the groundwater resource with different classes of degradation or improvement in a study area or province. Equation 7 was used with each cell score (replace *D*_*T*_with *S*_cell_) to compute the spatially weighted proportion of area within a study area, province, or the state having each class of cell score. For some figures and tables, the nine classes above were reduced by combining moderate and high concentrations for improving (classes 4 and 5) and degrading conditions (classes 7 and 8) or by combining degrading and improving with low concentrations (classes 6 and 7). Results for all nine classifications are provided in the supplemental material (Table S5).

### Redox, land use, and age classification

**Fig. 3:**
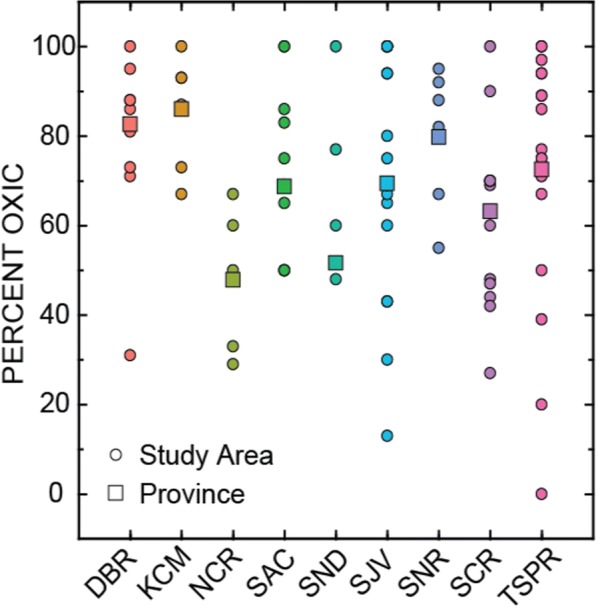
Percentage of oxic groundwater (dissolved oxygen ≥ 1 mg/L) in study areas (circles) and provinces (squares) in California. Hydrogeologic province abbreviations are defined in the text and Fig. 1

Dissolved oxygen (DO) is an essential measurement for determining redox in groundwater samples but it is not required for monitoring of groundwater for compliance purposes in the State of California. Therefore, redox conditions in groundwater resources used for public supply were estimated from PSWs sampled by GAMA-PBP only. Because about 20% of the GAMA-PBP PSWs with DO data did not also have data for other species required for more detailed redox classification, as used by McMahon and Chapelle (2008), a simplified classification was used: wells with DO ≥ 1 mg/L were classified as oxic, and wells with DO < 1 mg/L were classified as anoxic. The percentages of oxic and anoxic were computed for each study area and then the percentage oxic in each province was calculated as the area-weighted mean of the percentages in the study areas within the province. Statewide, about 73% of the groundwater resources used for public supply is oxic. While most provinces are predominately oxic, redox conditions can vary within provinces (Fig. 3). Anoxic groundwater is more likely to occur in aquifers with more abundant organic matter and older groundwater ages (more than several thousand years) because these conditions promote oxidation/reduction reactions (Fig. 3).

**Fig. 4:**
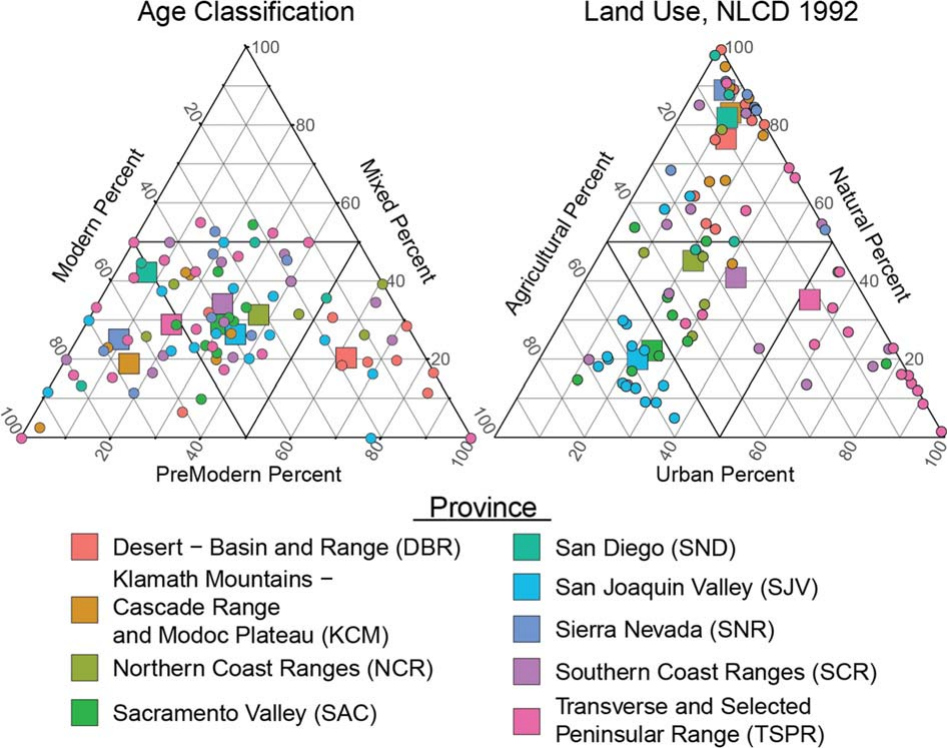
The percentage of different land use and groundwater age classes among study areas (circles) and hydrogeologic provinces (squares) in California

Land use in areas with PSWs was estimated for each study area as the average land use within a 500-m buffer around wells in the GAMA-PBP grid-well network. The grid-well network consists of one PSW in each grid cell of every study area (Fig. 1). Land use classes from the 1992 nationwide USGS National Land Cover Dataset (Nakagaki et al. 2007) were consolidated into three groups: urban, agricultural, and natural land uses (Johnson and Belitz 2009). Most of the area used for public supply in the SJV has agricultural land use; the majority in TSPR has urban land use; the majority in SNR, KCM, DBR, and SAN had natural land use; and SCR, NCR, and SAC have all three land use types roughly equal (Fig. 4).

The age of groundwater was classified as *Modern*, *PreModern*, or *Mixed* based on measurements of tritium (^3^H) and their relation to the history of ^3^H in precipitation [Michel et al. 2018], corrected for decay to the time of sampling [Lindsey et al. 2019]. *Modern* groundwater is water that was primarily recharged since 1950 and typically has ^3^H above 2 tritium units (TU). *PreModern* groundwater is water primarily recharged before 1950 (usually thousands of years before 1950) and typically has ^3^H below 0.3 TU. *Mixed* groundwater is water that has two (or more) components of water, one recharged after 1950 and another recharged before 1950 (usually thousands of years before 1950). *Mixed* groundwater typically has ^3^H between 0.3 and 2 TU and can occur more frequently in wells with long screens because they integrate large vertical segments of aquifer that can contribute water with contrasting or discontinuous recharge histories. The groundwater resource used for public supply in the DBR is predominately *PreModern*, the majority of groundwater resource used for public supply in SNR, KCM, TSPR, and SND is predominately *Modern*, and in SAC, SJV, SCR, and NCR, *Modern*, *Mixed*, and *PreModern* are roughly equally (Fig. 4).

## Results and discussion

### Statewide trends

Overall, about 69% (8866 of 12,926 wells with sufficient data) of PSWs in the State of California had at least one inorganic constituent with at least one statistically significant water-quality trend. About 87% of those wells had at least two constituents with trends indicating that trends tend to co-occur at individual wells, which is not unexpected since many water-quality constituents are interrelated. LTTs were the most commonly detected trend type and were detected at roughly 65% of wells. RTs, TRVs, and seasonal trends were detected at 57%, 20%, and 14% of wells tested, respectively.

#### Long-term trends

**Fig. 5:**
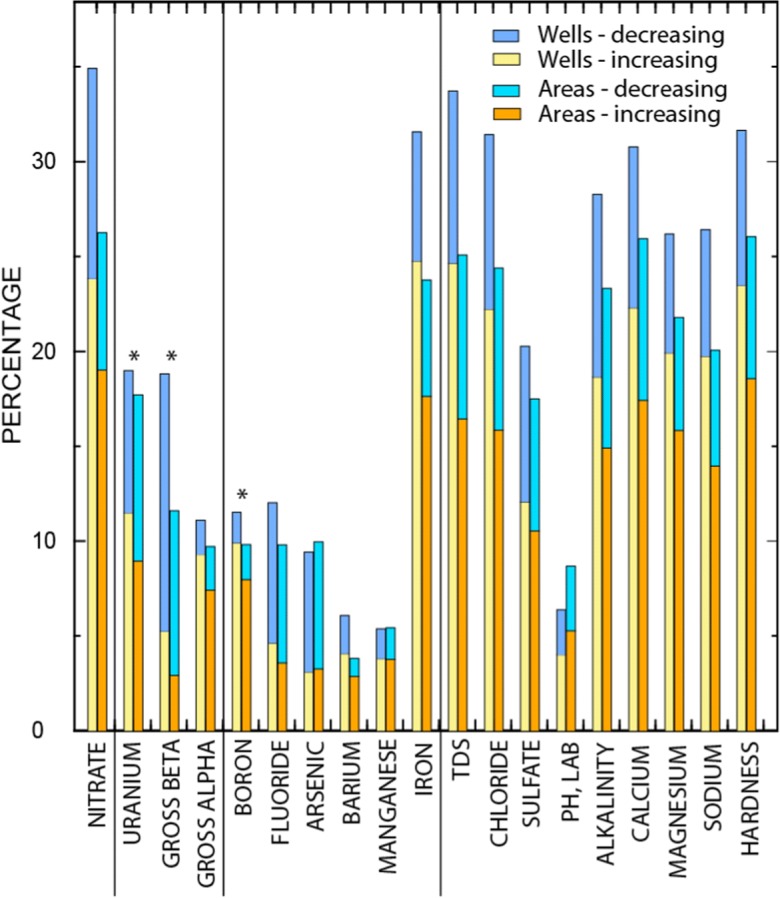
The percentage of wells and areas with long-term trends in concentrations (1974–2014) in the State Of California. The first bar of each constituent is the percentage of wells and the second bar is the percentage of area. Nineteen (shown) of 38 constituents had detections of long-term trends in 3% or more of the area. The percentage of wells or areas with increasing long-term concentration trends have yellow or orange bars and decreasing concentration trends have blue bars. Constituents marked with an asterisk had many fewer wells with sufficient data; therefore, results for those constituents may not be representative of statewide conditions

Statewide, spatially weighted results show that nitrate (26%), TDS (24%), and the major ions contributing to TDS (22–26%) had the highest percentages of area with LTTs (Fig. 5; Table S1). Spatially weighted results were frequently lower than detection frequencies of LTTs in wells but show similar constituent patterns (Fig. 5; Table S1). This comparison suggests that areas with changing groundwater quality conditions tend to have higher densities of wells. Nitrate was more frequently monitored than any other constituent. Most constituents had a sample population of about 7600 wells that could be tested for LTTs, whereas nitrate had about 12,300 wells (Table S1).

The radioactive constituents, uranium, radium, gross beta, and gross alpha, had areas with LTT of 0 to 18% (Table S1; Fig. 5). Uranium was not analyzed as frequently (~ 2600 wells) as gross alpha because uranium is not required for monitoring unless gross alpha is greater than 15 pCi/L. Consequently, gross alpha was used to assess uranium trends statewide because gross alpha activity is mainly from the activity of uranium in most oxic groundwater, and over 70% of the groundwater resources used by PSWs are oxic (Fig. 3). Gross beta and radium were analyzed even less frequently so the percentage of areas with uranium, gross beta, and radium trends are not reliable estimates of statewide trends (< 30% of gridded area) but can be important locally where they occur.

The trace elements—boron, fluoride, arsenic, barium, manganese, and iron—had areas with LTTs of 5 to 10% (Fig. 5). Arsenic and iron were the only constituents for which the percentage of area with LTTs was greater than the percentage of wells with LTTs. Eighteen constituents had LTTs in less than 3% of the area statewide: radium-226, chromium (total), selenium, aluminum, radium-228, lead, nickel, copper, mercury, antimony, beryllium, thallium, cadmium, nitrite, iodide, potassium, zinc, vanadium, silver, combined radium-226 + 228 (Table S1).

Overall, constituent concentration trends in wells and areas across the state are increasing (positive trends) more than decreasing (negative trends). Arsenic and fluoride were the only constituents for which decreasing concentration trends were more prevalent than increasing trends. Changes in constituent concentrations were generally low based on average Sen-slopes of LTTs (Table S1). For example, the average increase of nitrate concentrations statewide was about 0.03 mg/L per year (as nitrogen) in areas of positive trends and decreased about 0.01 mg/L per year in areas of negative trends.

#### Recent trends

**Fig. 6:**
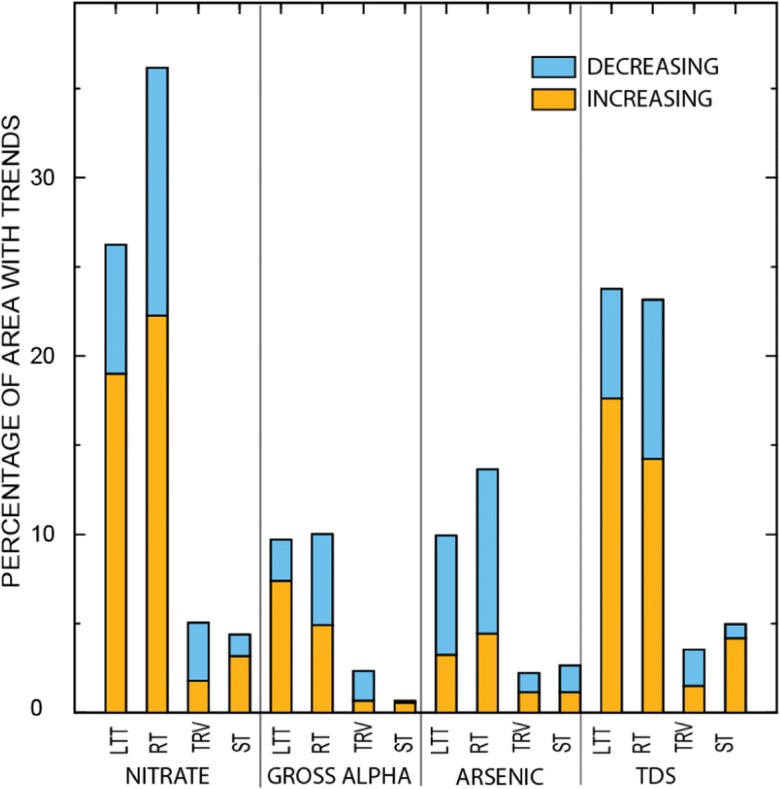
Comparison of areas with long-term (first bar), recent (second bar), reversing (third bar), and seasonal (fourth bar) trends for nitrate, gross alpha, arsenic, and TDS in public-supply wells in California. The percentage of the total area experiencing trends is the sum of the percentages of areas that have increasing concentration (red bars) and decreasing concentration (blue bars) trends

Recent trends (RT) were evaluated for data since the year 2000 (Table S2). In comparison to LTTs, nitrate and arsenic were the only constituents with more than a 1% increase in areas with recent trends (Fig. 6; Tables S1, S2). For all other constituents, the percentage of area with recent trends were within 1% of LTTs results (Tables S1, S2; Fig. 5). Because LTTs use the entire period of record, the number of wells and hence the areas of the state that were evaluated is slightly larger than the area evaluated using data since 2000. On average, the number of wells evaluated for each constituent using the LTT data was about 7600 wells (Table S1) and about 5700 wells (Table S2) using RT data. In addition, wells that have been abandoned or destroyed comprise about 3% of all significant LTTs (435 wells) whereas less than 0.4% (32 wells) had been abandoned that also had significant RTs. Wells that have had a history of contamination problems are often abandoned or destroyed. Consequently, the LTT results can reflect a broader area with poorer quality of water in aquifers used for public supply across the state whereas the RT results reflect areas of current use where groundwater quality is better for most constituents.

**Table 3:** Percentage of RT-based cell classifications in percent for nitrate, total dissolved solids, gross alpha, and arsenic in nine hydrogeologic provinces of California. The percentage of cells that have improving (Imp.) conditions with moderate and high concentrations, cells that have either improving or degrading (Deg.) conditions with low concentrations (less than half the benchmark), cells that have degrading conditions with moderate and high concentrations, and cells that have trends with similar magnitudes but opposite directions or indeterminate are given for each constituent. Provinces in which the percentage of cells with low and degrading conditions is greater than the percentage of cells with low and improving conditions are formatted as italicized text. Percentage of cell areas that are changing or could not be tested are not included but can be seen in Fig. 7

Hydrogeologic province	Nitrate				Total dissolved solids				Gross alpha				Arsenic			
	Hi or Mod Imp.	Low Imp., Deg.	Hi or Mod Deg.	Ind.	Hi or Mod Imp.	Low Imp., Deg.	Hi or Mod Deg.	Ind.	Hi or Mod Imp.	Low Imp., Deg.	Hi or Mod Deg.	Ind.	Hi or Mod Imp.	Low Imp., Deg.	Hi or Mod Deg.	Ind.
Desert—basin and range (DBR)	1.3	*36.6*	2.7	2.1	3.0	*17.2*	2.5	0.2	3.0	8.8	1.7	0.3	3.8	4.0	5.2	0.1
Klamath Mountains—Cascade Range and Modoc Plateau (KCM)	0.3	*8.5*	0.5	0.5		*24.3*					0.5		0.9	4.2	0.9	
Northern Coast Ranges (NCR)	1.2	17.9	1.0	2.6	0.1	*11.6*	1.2	2.5		*1.2*			8.7	2.9	0.6	
Sacramento Valley (SAC)	4.9	*33.9*	3.7	2.1	2.5	*8.0*	1.0	1.0		*2.4*			7.9	2.7	4.9	0.3
San Diego (SND)	1.6	*19.9*	1.5	1.6	5.7	*13.4*	8.9	3.1	4.9	11.5	4.4	0.7	2.8	*3.7*		
San Joaquin Valley (SJV)	5.1	*29.1*	10.8	1.2	1.7	*19.1*	2.9	0.9	1.9	6.4	2.9	0.4	8.7	8.2	3.6	0.6
Sierra Nevada (SNR)	1.0	*13.3*	0.8	1.3	0.1	*15.2*		1.2	6.8	2.3	0.9	0.0	6.2	*2.5*	0.1	0.1
Southern Coast Ranges (SCR)	4.9	*30.4*	5.9	2.9	6.6	*13.2*	4.8	1.4	0.9	*6.1*	0.2	0.4	2.1	4.4	1.6	0.1
Transverse and Selected Peninsular Range (TSPR)	10.1	*32.3*	10.3	2.1	6.0	*22.2*	10.3	2.4	2.6	8.5	1.1	1.0	3.2	4.1	0.2	0.1
Statewide	3.7	*25.5*	5.3	1.7	2.5	*16.3*	3.1	1.3	2.9	*5.3*	1.5	0.3	6.2	4.7	2.4	0.3

#### Trend reversals

Statewide, nitrate, gross alpha, TDS, sodium, chloride, sulfate, and pH all had significant trend reversals in more than 2% of areas used for public supply in the state (Table S3; Fig. 6). Most of the trend reversals were negative indicating trends reversed from an increasing trend to a decreasing trend. However, pH had more increasing trends than decreasing trends, which could indicate a greater contribution of *PreModern* groundwater from deep parts of the groundwater system where pH is usually higher.

#### Seasonal trends

Statewide, nitrate was the only constituent with sufficient data to test for seasonal trends in more than 50% of areas used for public supply (Table S4; Fig. 6). About 4.4% of the assessed area showed seasonal trends for nitrate. TDS, calcium, chloride, sodium, and hardness had seasonal trends in greater than 5% of the assessed area, but less than 30% of the area could be assessed. These results indicate that most wells do not have trends that are masked by seasonal concentration differences statewide, but seasonal trends likely have greater occurrence in a few provinces or local areas. Statewide, Mann-Whitney test for differences between seasonal concentrations found that about 20% of wells had some difference between summer and winter concentrations and about half the concentrations were lower in the winter than in the summer.

### Improving and degrading groundwater quality within hydrogeologic provinces

**Fig. 7:**
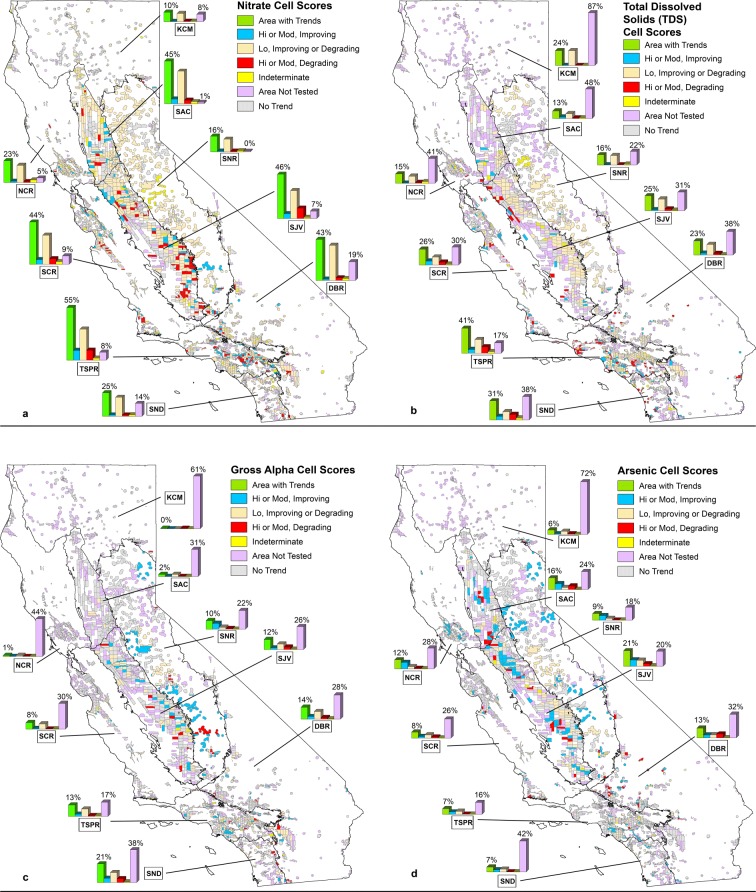
Classifications and percentages of RT-based cell scores for **a** nitrate, **b** total dissolved solids, **c** gross alpha, and **d** arsenic in nine hydrogeologic provinces of California. The bar charts give the percentage of cells in a province that have improving conditions (decreasing concentrations) with moderate to high (blue) concentrations, improving or degrading conditions with low (beige) concentrations and the percentage of cells with degrading conditions (increasing concentrations) with moderate to high concentrations (red). The percentage of cells that have any kind of trend (green), that are indeterminate because of an equal number of positive and negative trends scores for wells within the cell (yellow), and where trends could not be evaluated because of insufficient data (purple) are also provided

Recent trends for nitrate, TDS, arsenic, and gross alpha were detected in about 36, 23, 14, and 10% of areas used for public supply in the state (Table S2). These constituents were the most commonly detected constituent trends and represent a range of constituent types: nutrients, radioactive, trace element, and major ion chemistry. Classifications of cell scores were aggregated for each hydrogeologic province (Table 3) and mapped (Fig. 7) to show the distribution of trends and concentrations across the state. Overall, the percentages of area with trends (improving or degrading) were greatest in the TSPR, SJV, and SCR provinces. In contrast, the KCM, NCR, SNR provinces had the lowest percentages of areas with changing conditions (Table 3).

The DBR and SND provinces also had a significant percentage of area with trends and high concentrations but these areas also had a large percentage of unassessed areas (purple bar in Fig. 7). Provinces with more than 25% of unassessed areas most often were in the DBR, KCM, and SND provinces so results for these areas may not be accurate provincially but can help identify water-quality issues locally. These provinces tend to have many non-community systems, which are less frequently monitored for constituents other than nitrate.

#### Nitrate

Of all constituents tested, nitrate had the largest percentage of area with improving or degrading conditions at moderate to high concentrations (Fig. 7). The percentage of area that could not be tested for nitrate trends was lowest of all constituents tested (Table S5), which indicates the results for nitrate provide meaningful statewide and provincial estimates of improving and degrading conditions.

Areas where nitrate was improving or degrading and had either moderate or high concentrations were most prevalent in the SCR, SJV, and TSPR provinces at 11%, 16%, and 20% of the total area, respectively (Table 3). These provinces have the three conditions required for trends and high concentrations to occur: a change in the input of nitrate at the land surface (generally due to land use change), wells that tap groundwater with age distributions that include the period of nitrate input (Fig. 4), and oxic conditions to preserve the nitrate (Fig. 3).

**Fig. 8:**
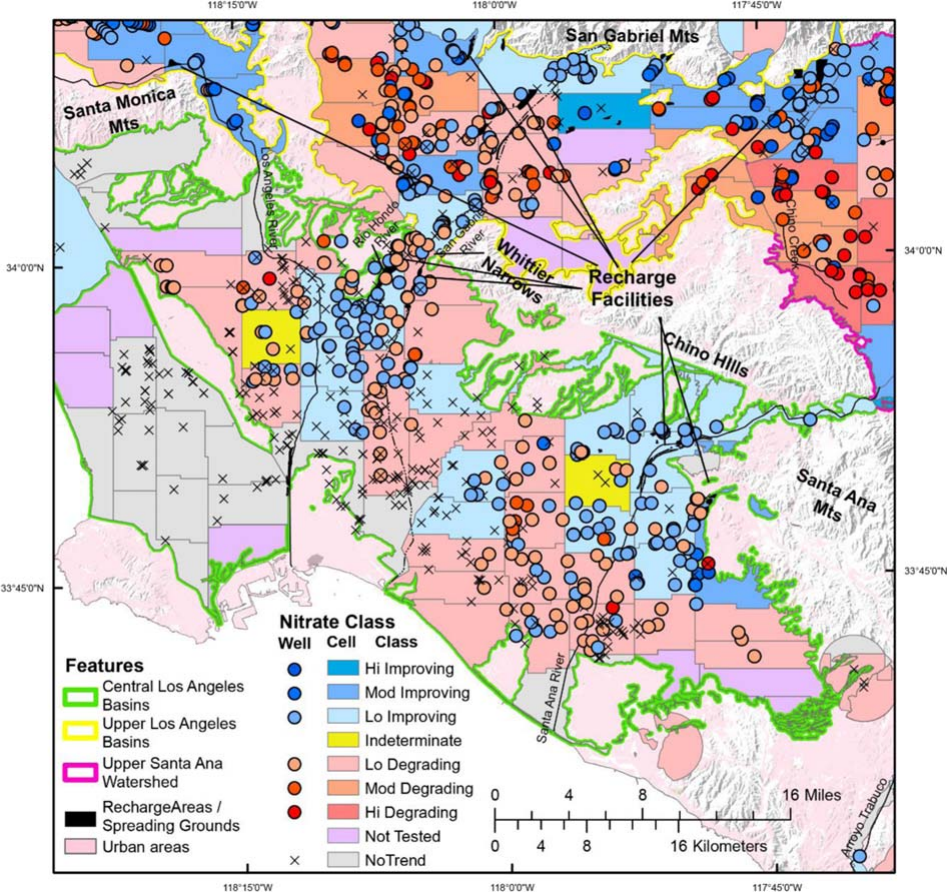
Nitrate well and cell scores for areas in the Transverse and Selected Peninsular Ranges (TSPR) based on RT results. Blue areas on the map indicate decreasing concentration trends while red areas indicate increasing concentration trends. Areas colored light blue and light red have nitrate concentrations below half the MCL (45 mg/L as nitrate or 10 mg/L as nitrogen), while darker colors indicate nitrate concentrations above half the MCL and above the MCL. Indeterminate areas are colored yellow and contain wells with trends in opposite directions and equal in magnitude (cancel out). Areas colored gray do not have wells with trends and areas colored purple were not tested for trends because there were no wells located in the cells or wells within that cell did not have enough data to evaluate trends

**Fig. 9:**
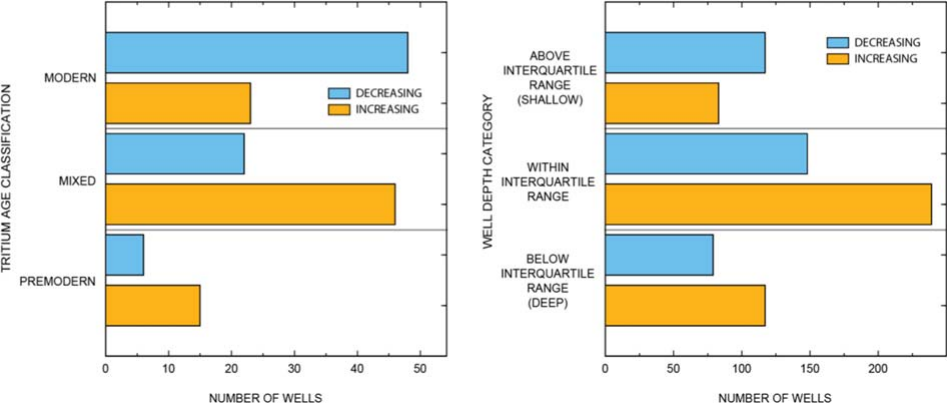
Bar graphs of the number of wells in the Transverse and Selected Peninsular Ranges (TSPR) with increasing and decreasing nitrate trends for different well construction and age classifications

In the TSPR, PSWs mostly tap *Modern* or *Mixed* groundwater with oxic conditions (Figs. 3 and 4) derived from water that infiltrated on spreading grounds at recharge facilities or that recharged along a mountain front (Fig. 8). Land use is currently dominated by urban (Fig. 4) land but had been farmed in the past (Scott 1977; Hamlin et al. 2005). Recharge before agricultural activities had low nitrate, followed by high nitrate in recharge beneath agricultural land, and finally lower nitrate in recharge following urbanization (Reichard et al. 2003). The percentage of areas with moderate to high nitrate concentrations and either improving or degrading conditions in the TSPR was similar (Table 3). These trends likely reflect increasing nitrate concentrations in deeper, *PreModern* groundwater catching the change of water recharged beneath agricultural land, while shallower wells with *Modern* groundwater are catching the more recent recharged beneath urban land and have decreasing nitrate concentrations (Fig. 9). In addition, wells located closer to areas of recharge are more likely to have decreasing concentration trends while groundwater downgradient that was recharged long before 1950 (*PreModern*) tend to have increasing nitrate trends (Fig. 9). These findings are consistent with the observation that the TSPR province has the most TRVs.

In contrast to the TSPR, land use in the SJV is still dominated by agricultural land and the groundwater tapped by PSWs is on average older than in the TSPR (Fig. 4), so the dominance of increasing nitrate concentration trends in the SJV suggests that the recharge beneath agricultural land with high nitrate is reaching the PSWs now and encompassing a greater proportion of the water captured by these wells. These trends are also consistent with observations that domestic wells, which are generally shallower and have younger water than PSWs, have a greater proportion of wells with high nitrate concentrations than do PSWs in the SJV (Burow et al. 2013; Shelton and Fram 2017). The lower occurrence of high/moderate concentrations and nitrate trends in the other provinces reflects an absence of one or more of the three necessary conditions mentioned above or that the three conditions are not as prevalent.

#### TDS, gross alpha, arsenic

TDS concentrations have generally been increasing in groundwater more than it has been decreasing statewide but overall TDS trends occur mainly in wells with low concentrations (Table 3). This suggests that long-term salinization of the groundwater resources used for public supply is occurring across the state. In the TSPR, about 29% of the area used for public supply has degrading TDS conditions although about 19% of this area has low concentrations (Table S5). TDS is not as frequently monitored as the other four constituents and provinces with more than 25% of the area not tested cannot be adequately assessed without additional monitoring (Fig. 7; Table 3).

At the statewide level, the slope of TDS trends was correlated with the slope of nitrate trends in wells where the trends co-occur (Spearman’s rho = 0.43, *p* value < 0.001). This suggests that increasing TDS concentrations may partly result from agricultural practices. However, this correlation is heavily influenced by places where both nitrate and TDS trends frequently co-occur, such as the SJV, TSPR, and SCR. For example, concentrations of nitrate and TDS were correlated in SJV (0.47) but were not correlated in SND. In areas where relations between TDS and nitrate are absent, relations with other major ions may help identify TDS sources.

Major ion (calcium, magnesium, sodium, bicarbonate, sulfate, chloride) trends are often correlated to TDS trends but the strength of correlations between Sen’s slopes may depend on the source of TDS. In SND, 50 wells had increasing TDS concentrations while 28 had decreasing TDS concentrations. The Sen’s slope for magnesium and chloride were the most strongly correlated cation (sodium, calcium, and magnesium) and anion (sulfate, chloride, bicarbonate) with TDS in SND. TDS trends also were frequently co-detected with trends of sodium, calcium, chloride, and sulfate suggesting that many TDS trends were associated with a brackish groundwater source or groundwater derived from the dissolution of marine evaporite sediments. In places where evaporation or seawater intrusion are sources of TDS trends, it might be expected that correlations with sodium and chloride are strongest.

The TSPR, SCR, and SND provinces had the largest areas of trends with moderate to high TDS concentrations (Fig. 8; Table 3). All three provinces have coastal connections and support or previously supported agricultural farming. Consequently, it is possible that sources of TDS trends such as seawater intrusion, brackish water extraction, agricultural applications of soil amendments and fertilizers, or evaporative concentration of applied irrigation or recharge, could vary locally within these provinces.

Gross alpha trends with moderate to high concentrations occurred in 4.7, 4.8, 7.7, and 9.3% of areas in the DBR, SJV, SNR, and SND provinces, respectively (Table S5). Nearly 40% of the area in SND could not be tested for gross alpha trends so the percentage may not be accurate at the province scale. Most of the moderate to high gross alpha concentrations in the SNR were improving (decreasing concentrations) while most of the moderate to high concentrations of gross alpha in the SJV and DBR provinces were associated with degrading conditions (increasing concentrations), about 2.9 and 1.7%, respectively. Statewide, gross alpha trends were strongly correlated to uranium trends in the same well (Spearman’s rho = 0.65, *p* value < 0.001), which indicates that uranium is a significant contributor to gross alpha in most groundwater. High or moderate concentrations of gross alpha and uranium were frequently found to occur in the SJV in the past (Jurgens et al. 2010) and were linked to increases in alkalinity, which can complex uranium and make it more mobile in the subsurface. Statewide, gross alpha trend slopes were correlated with alkalinity trend slopes (Spearman’s rho = 0.44, *p* value < 0.001), indicating gross alpha increases are frequently linked with increases in alkalinity and uranium in wells. Additional monitoring of gross alpha, TDS, and major ions may help identify other wells and areas experiencing gross alpha increases due to alkalinity.

While most trends for nitrate, TDS, and gross alpha were associated with low concentrations, most arsenic trends were associated with moderate or high concentrations (8.6%) statewide (Table 3). Arsenic concentration trends were most often decreasing in groundwater across the state. Provinces with moderate to high concentrations and improving conditions comprised about 4% or more of areas in the DBR, NCR, SAC, SJV, and SNR while provinces where impaired (high concentrations) and degrading conditions comprised more than 3% in DBR, SAC, and SJV (Table 3). Given the occurrence of arsenic trends in the DBR, additional sampling for arsenic would permit a better assessment of arsenic conditions in approximately 34% of the area that could not be tested. In addition, co-detections of trends with arsenic did not commonly occur in the DBR, which may be the result of infrequent sampling for major ions when a well belongs to a non-community system.

Arsenic tends to be mobilized in groundwater with high pH or reduced geochemical conditions because these conditions favor the release of arsenic from sorption sites on iron-oxyhydroxides coatings on sediments (Smedley and Kinniburgh 2002). The median pH of water from wells where RTs were tested was high in SAC and SJV at 7.8 and 7.9, respectively. The percentage of *PreModern* groundwater was also high in SAC and SJV (Fig. 4). Groundwater with long residence times allow for more water-rock reactions to occur and typically leads to higher pH and reduced geochemical conditions (low DO).

In SAC, arsenic trends were correlated with pH trends (Spearman’s rho = 0.35, *p* value = 0.09), whereas manganese was more closely associated with arsenic trends in the SJV (Spearman’s rho = 0.48, *p* value = 0.05). Because most arsenic concentration trends in these areas are decreasing, arsenic likely is decreasing in response to lower pH and manganese concentrations (more oxic conditions). In addition, decreasing arsenic concentrations were correlated with increasing nitrate concentration trends in SAC and SJV (SAC rho = − 0.38, *p* value = 0.002; SJV rho = − 0.40, *p* value < 0.001). This suggests that more areas are experiencing a greater contribution of water with more oxic conditions that promote arsenic immobilization. As yearly recharge of oxygenated groundwater is repeated, wells that are screened across long segments of aquifer or have typically extracted geochemically reduced groundwater in the past may capture an increasing portion of younger, more oxic, groundwater over time.

#### Cell score limitations

Scores were computed constituent by constituent such that one constituent may exhibit improving conditions while another constituent may indicate degrading conditions. As such, the approach developed in this paper did not evaluate the whole quality of the groundwater resource. It is possible the method could be used to assess improving and degrading areas more generally by aggregating all constituent concentration and trend scores. Results from this adjustement would tend to illuminate areas where high concentrations of any constituent occur in the state.

Trend scores were computed using the Sen’s slope estimate of the trend. This measure of change assumes a linear increase or decrease in concentrations, but most trends display nonlinear rates of change. Therefore, the Sen’s slope estimate may not give accurate predictions of concentrations at a single site. In addition, the Sen’s slope for RTs is the set of data with the largest magnitude of change so cell scores may be biased areas towards more extreme degrading or improving scores. Areas with many wells will tend to moderate extreme rates of change in the aggregation process.

The well and cell scores presented in Fig. 8 contain all possible classes from the scoring metric developed in this paper and is an example of the detail seen locally that is difficult to convey visually statewide. The aquifers underlain by the areas shown in Fig. 8 can be separated by confining units that may restrict the vertical migration of groundwater flow (Reichard 2003; Hamlin et al. 2002). Some aquifer units may have improving while other units may have degrading conditions. Because multiple units can supply water to consumers, cell scores can be biased towards one unit with trends over other units that do not display any trends when scores are averaged across aquifer units, such that the resulting score may not capture the full three-dimensional nature of the system. This underscores the importance of understanding where in the groundwater system trends are occurring and what type of water trends are associated with because concentration trends may be rising in some wells while falling in others (Fig. 9).

As a final evaluation of this method, trend results for cells were compared to regional Mann-Kendall tests for nitrate trends in 1546 cells. Overall, trend scores and the regional MK results were similar and trend directions agreed in 83% of cells where regional MK results were significant (571 of 686 cells). Trend scores identified 342 additional cells with trends based on individual well results. Most of these cells had trend detection frequencies in wells of less than 50%, suggesting the regional MK test can fail when most wells in an area do not have a trend. In cells with low frequencies of trend detections, the trends can be useful for identifying an oncoming problem that could otherwise go unnoticed until most wells are affected by contamination.

### Well trend web map

The semi-automated routine described in the methods section looked at time series from nearly 13,000 wells and 38 constituents. This routine generated over 500,000 results, which makes it difficult to condense results into data files and summarize important findings. Therefore, a website was created that presents the individual results by constituent and trend type for every well that was tested (Dupuy et al. 2019). The GAMA Trends Web Map website (https://ca.water.usgs.gov/projects/gama/public-well-water-quality-trends/) allows users to see trends at different scales across the state, view graphs of data and trends, and links to the datasets for each individual well.

## Conclusions

The grid-based scoring metric was used to identify areas of improving and degrading groundwater quality conditions in hydrogeologic provinces in the State of California. This method required the creation of a network of equal-area grid cells that cover wells that supply groundwater for public drinking water in the state. The network of cells was used to aggregate constituent concentrations and trend scores for individual wells to multiple spatial scales, beginning upward in area, from cells to study areas to hydrogeologic provinces to the entire state. The trend scores give similar results to regional MK tests but include additional areas where detections of trends in wells is less frequent but may serve as an early indicator of water-quality issues in an area.

Results from this method showed that concentrations of nitrate (36%), gross alpha (10%), arsenic (14%), TDS (23%), and the major ions that contribute to TDS (19–28%) were the most frequently detected trends in areas used for public-supply statewide. For these constituents, the TSPR, SJV, SAC, and SCR hydrogeologic provinces had the largest percent of areas (on average) experiencing trends at 32, 26, 24, and 23%, respectively. The main limitation of computing accurate areal proportions of trends was the lack of data in provinces with large rural and non-community systems, such as the DBR, KCM, NCR, SND, and SNR. Additional sampling for major ions and constituents with non-enforceable benchmarks would improve the assessment statewide and enable better understanding of why water quality is changing in those areas.

Current and historical applications of nitrogen fertilizers have led to widespread occurrence of nitrate trends with elevated concentrations (moderate to high) in many areas used for public supply throughout the state. In areas where agricultural land has been largely urbanized, like the TSPR, a significant portion of area had improving concentrations. Thus, land use change accompanied with low nitrate recharge has remediated some areas where groundwater once was impaired. Although significant urbanization of agricultural land in places like the SJV is unlikely to happen soon, winter diversions of excess surface water onto agricultural fields may lessen the impact of nitrate loading beneath agricultural fields during the summer growing season.

Arsenic was the only constituent with more decreasing concentration trends (9.2%) than increasing trends (4.4%) statewide. Arsenic trends were most often associated with moderate to high concentrations and most arsenic concentrations were improving statewide. Correlations between arsenic trends and nitrate trends in SAC and SJV provinces suggest that many wells are capturing an increasing contribution of more oxic groundwater with lower arsenic that is being driven downward by repeated cycles of recharge and groundwater pumping in agricultural areas in these provinces.

Finally, the groundwater-quality trend results could be enhanced by coupling water-level monitoring with water-quality data. Water-level information would provide a vital link to understanding long-term water-quality changes in response to drought, climate change, and groundwater management decisions.

## Electronic supplementary material

ESM 1 (DOCX 236 kb)
