# Supplementary material for: Identifying areas of degrading and improving groundwater-quality conditions in the State of California, USA, 1974–2014
Source: Environ Monit Assess. 2020 Mar 25;192(4):250. doi: 10.1007/s10661-020-8180-y (PMC7096367; doi:10.1007/s10661-020-8180-y)
Supplement: Supplementary file 1 — (DOCX 236 kb) [file 10661_2020_8180_MOESM1_ESM.docx]

# Supporting information

## Identifying Areas of Degrading and Improving Groundwater-Quality Conditions in the State of California, 1974—2014

#### By Bryant C. Jurgens^1^, Miranda S. Fram^1^, Jeffrey Rutledge^1^, George L. Bennett^1^

U.S. Geological Survey, California Water Science Center, Sacramento 95819, CA, United States

Number of pages: 13

Number of Figures: 4

Number of Tables: 5

1. Count of the number of public-supply wells with data for one or more inorganic constituents in the California State Water Resources Control Board – Division of Drinking Water (SWRCB-DDW) electronic database (SWRCB-DDW, 2016), and additional samples collected by the California Groundwater Ambient Monitoring and Assessment Program Priority Basin Project (GAMA-PBP; California State Water Resources Control Board, 2018), 1974 to 2014. The increase in the number of wells between 1974 and 2002 was mostly due to an increase in electronic reporting of water-quality data. The number of public-supply wells also increased during this period.

## Effect of Equal Values on Trend Tests

Equal values or ties in a dataset can make it difficult to evaluate statistical significance of Kendall’s rank correlation (Amerise and Tarsitano, 2016). Water-quality data often contains ties because of frequent occurrences of non-detections at common reporting levels and of detected concentrations caused by analytical precision and rounding. In addition, the truncation level applied to non-detections and detections below the truncation level can introduce equal values. Mann-Kendall rank correlations were computed using the SciPy package in Python, which uses Kendall’s tau-b to account for ties:

| $\tau_{b}=\frac{P-U}{\left[ \left( P+Q+Y_{o} \right)\left( P+Q+X_{o} \right) \right]^{\frac{1}{2}}}$ | (1) |
| --- | --- |

Where,

$\tau_{b}$ is the Kendall tau-b statistic which accounts for ties in data

$P$ is the number of concordant pairs

$Q$ is the number of discordant pairs

$Y_{o}$ is the number of pairs not tied in Y

$X_{o}$ is the number of pairs not tied in X

While this correction for tau is commonly used, exact p-values cannot be computed using the SciPy algorithm when ties exist [PSF, 2019]. The SciPy algorithm for Kendall’s tau-b calculates an approximate p-value.

In order to identify how p-values might be affected, a comparison was made between p-values determined from Kendall’s tau-b and exact p-values computed using the R-package pvrank (Amerise et al., 2016). The ‘pvrank’ package can account for ties in a dataset using different methods. This comparison revealed that p-values using Kendall’s tau-b method were below a significance level (α) of 0.1 when the number of unique values in a dataset (number of data minus number of ties) was less than 4 and Kendall’s tau-b p-values became increasingly lower as the number of ties increased (Figure S2). Exact p-values computed using the Woodbury methods showed that when ties were present in the example in Figure S2, most p-values were above 0.1 and not significant. Because an ordered set of four increasing values (e.g., 1, 2, 3, 4) has an exact p-value of 0.083 below the significance level of 0.1, datasets which contained three unique values or less were recorded as not significant. In addition, there may be other situations where ties could cause Kendall’s tau-b to be significant. Since Sen’s slope will be zero when most values are ties in a set of data, the criteria for statistical significance was made more stringent by requiring Sen’s slope not be zero in addition to requiring 4 or more unique values.


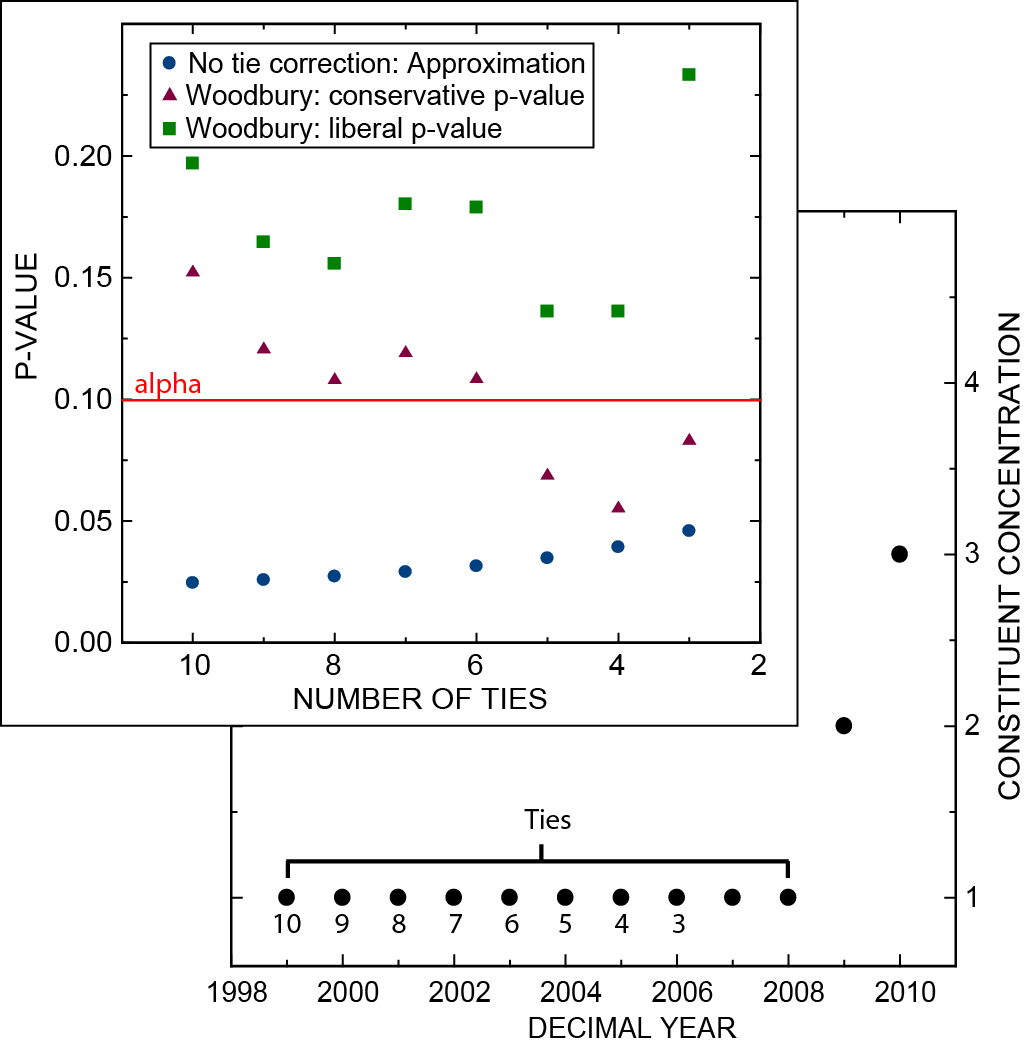


1. Ties affect the computed p-value of Mann-Kendall’s rank correlation. The bottom graph shows a series of data points with ties and two increasing data points. Mann-Kendall rank correlations are positive and p-values without corrections for ties are within the trend acceptance criteria (significance level = 0.1). However, exact p-values computed with corrections for ties (Woodbury) show the actual p-value is frequently above 0.1 and not significant.

## Data Processing

Inorganic water-quality data from the California State Water Resources Control Board – Division of Drinking Water (SWRCB-DDW) [SWRCB-DDW, 2016] and California Groundwater Ambient Monitoring and Assessment Program Priority Basin Project (GAMA-PBP) [Jurgens et al., 2018] were compiled and loaded into a database. Because irregular (more frequent and less frequent) temporal sampling can introduce biases that can affect the significance of trend results (Figure S1), the raw water-quality data was processed according the following steps:

Data were screened to the most common detection level such that data below the screening level were recoded to the screening level and non-detections above the screening level were removed from the dataset,

1. data with improper dates or null water-quality results were removed from the dataset,
2. the median value was used for constituents that had more than one result on a single date,
3. and data was binned into summer and winter seasons of each year resulting in at most two dates and sample results per year by computing the median date and concentration of each season. Summer was defined as May 1^st^ thru October 31^st^ of each year.

These procedures generally reduce the effects of serial correlation caused by frequent sampling over short time intervals (Figure S3) but does not lessen serial correlation caused by long gaps between samples (Figure S4).


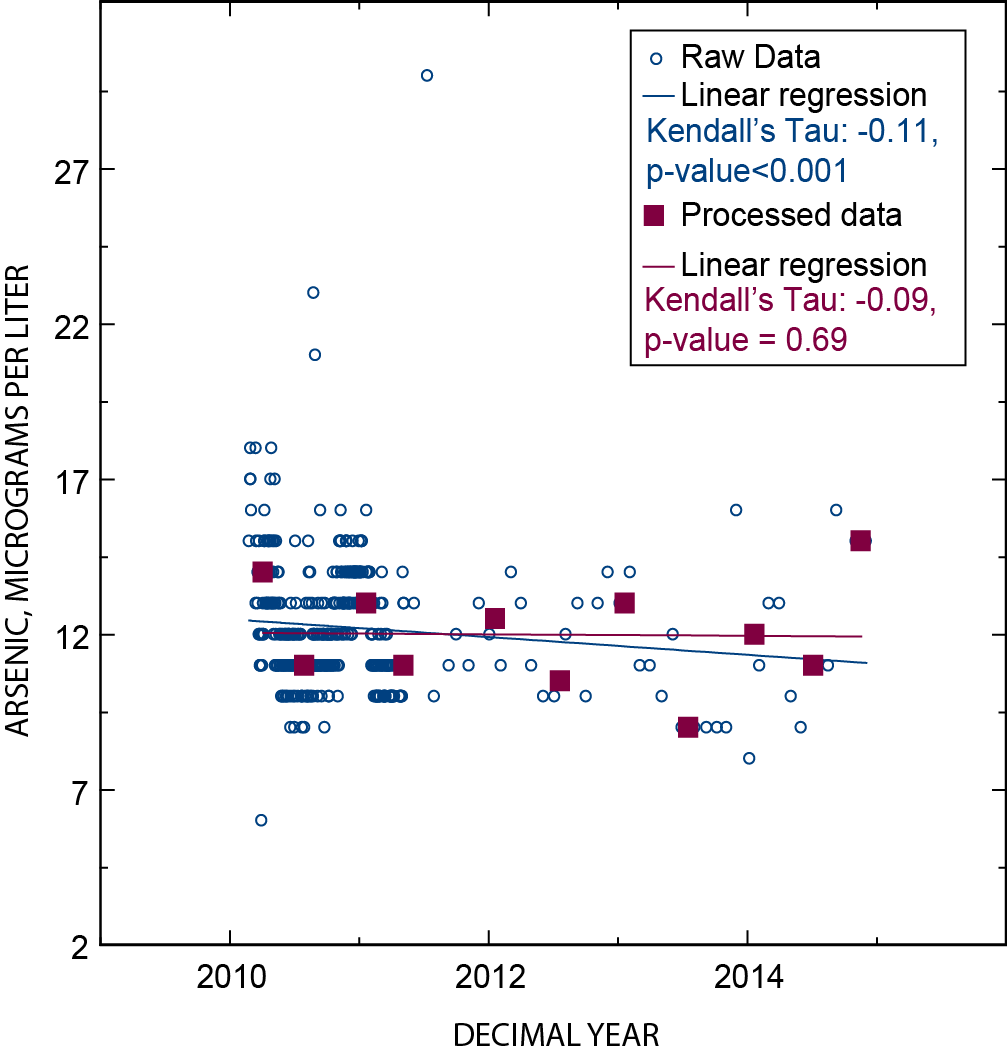


1. Irregular sampling patterns can affect Mann-Kendall rank correlations. This graph shows that frequent sampling in 2010 and 2011 causes a significant, negative trend to be detected. To remove the serial correlation, data were collapsed into median concentrations for each half-year season. In this case, trends were not detected using the processed data. The effect of the data pre-processing on trend calculations can vary based on the raw dataset. In some cases, the trend correlations can be strengthened.


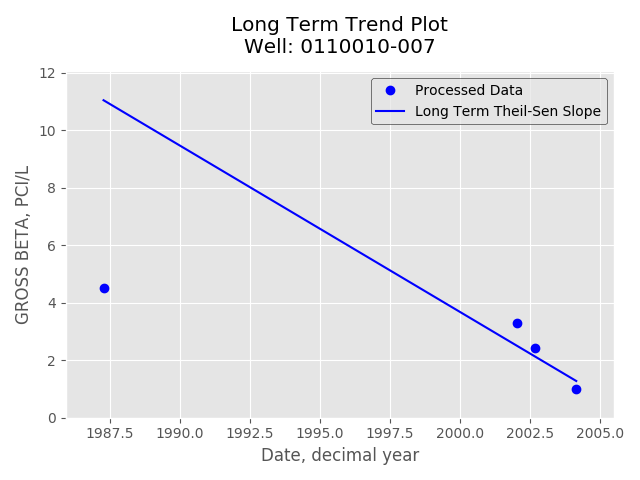


1. An example of serial correlation caused by data gaps.

# References Cited

Amerise I. L., Marozzi M., & Tarsitano A. (2016). pvrank: Rank Correlations. R package version 1.1.1. <http://CRAN.R-project.org/package=pvrank>.

Amerise I. L., Marozzi M., & Tarsitano A. (2015) Correction methods for ties in rank correlations. Journal of Applied Statistics 42 (12):2584-2596

California State Water Resources Control Board – Division of Drinking Water (SWRCB-DDW). (2016). EDT Library and Water Quality Analyses Data and Download page; <https://www.waterboards.ca.gov/drinking_water/certlic/drinkingwater/EDTlibrary.shtml>

California State Water Resources Control Board. (2018). GAMA – Groundwater Ambient Monitoring and Assessment Program Website, accessed February 21, 2018 at: <https://www.waterboards.ca.gov/gama/geotracker_gama.shtml>

PSF. (2019). Python Software Foundation. Python Language Reference, version 3.7. Available at http://www.python.org

# Supplemental Tables

For each table below, the percentage of wells is based on the total number of wells of each constituent listed in table 1. The percentage of gridded area is based on the total gridded area in California, 105,312.2 km^2^.

1. Long-term water-quality trends in public-supply wells in the State Of California (all available data: 1970 – 2014).

| Long-term Trends (LTT) | | | | Significant Trends | | | Positive Trends | | Negative Trends | |
| --- | --- | --- | --- | --- | --- | --- | --- | --- | --- | --- |
| Constituent | Units | Percent of wells tested | Percent of gridded area tested | Percent of tested wells with a trend | Percent of gridded area with a trend | Avg. Sen slope of tested area units/yr x 100 | Percent of gridded area with a trend | Avg. Sen slope of tested area units/yr x 100 | Percent of gridded area with a trend | Avg. Sen slope of tested area units/yr x 100 |
| **Nutrients** |  |  |  |  |  |  |  |  |  |  |
| Nitrate | mg/L as N | 79.6 | 77.7 | 34.9 | 26.2 | 2.4 | 19.0 | 3.3 | 7.2 | -1.0 |
| Nitrite | mg/L as N | 65.2 | 69.4 | 0.0 | 0.0 | 0.0 | 0.0 | 0.0 | 0.0 | 0.0 |
| **Radioactive constituents** | |  |  |  |  |  |  |  |  |  |
| Gross alpha | pCi/L | 65.9 | 61.7 | 11.1 | 9.7 | 1.5 | 7.4 | 2.4 | 2.3 | -0.8 |
| Gross beta | mg/L | 16.8 | 5.7 | 18.7 | 11.3 | -0.6 | 2.7 | 0.2 | 8.7 | -0.8 |
| Radium 226 | pCi/L | 16.0 | 9.3 | 0.8 | 2.4 | 0.0 | 0.20 | 0.0 | 2.2 | 0.0 |
| Radium 228 | pCi/L | 14.4 | 18.0 | 1.1 | 0.9 | 0.0 | 0.1 | 0.0 | 0.9 | 0.0 |
| Radium 226 + 228 | pCi/L | 7.5 | 2.1 | 0.0 | 0.0 | 0.0 | 0.0 | 0.0 | 0.00 | 0.0 |
| Uranium | pCi/L | 36.2 | 31.1 | 18.9 | 17.6 | 0.6 | 8.9 | 1.5 | 8.70 | -0.9 |
| **Trace elements** |  |  |  |  |  |  |  |  |  |  |
| Aluminum | ug/L | 62.2 | 60.7 | 0.70 | 0.90 | 1.1 | 0.40 | 3.1 | 0.40 | -2.0 |
| Antimony | ug/L | 60.9 | 59.0 | 0.00 | 0.00 | 0.0 | 0.00 | 0.0 | 0.00 | 0.0 |
| Arsenic | ug/L | 64.1 | 63.3 | 9.40 | 9.90 | -0.4 | 3.30 | 1.1 | 6.70 | -1.5 |
| Barium | ug/L | 62.5 | 61.3 | 6.10 | 3.80 | 7.9 | 2.90 | 11.3 | 1.00 | -3.4 |
| Beryllium | ug/L | 60.9 | 58.8 | 0.00 | 0.00 | 0.0 | 0.00 | 0.0 | 0.00 | 0.0 |
| Boron | ug/L | 29.2 | 24.6 | 11.50 | 9.80 | 42.1 | 8.00 | 52.7 | 1.80 | -10.5 |
| Cadmium | ug/L | 62.3 | 60.9 | 0.00 | 0.00 | 0.0 | 0.00 | 0.0 | 0.00 | 0.0 |
| Chromium (total) | ug/L | 62.6 | 61.5 | 1.30 | 1.00 | 0.1 | 0.60 | 0.2 | 0.40 | -0.1 |
| Copper | ug/L | 60.8 | 58.4 | 0.00 | 0.00 | 0.0 | 0.00 | 0.0 | 0.00 | 0.0 |
| Fluoride | mg/L | 60.5 | 61.7 | 12.00 | 9.80 | 0.0 | 3.60 | 0.1 | 6.20 | -0.1 |
| Iron | ug/L | 53.0 | 56.0 | 6.40 | 8.70 | -1.0 | 5.30 | 1.2 | 3.40 | -2.2 |
| Lead | ug/L | 58.2 | 53.8 | 0.10 | 0.00 | 0.0 | 0.00 | 0.0 | 0.00 | 0.0 |
| Manganese | ug/L | 60.1 | 60.1 | 5.40 | 5.40 | 21.8 | 3.80 | 33.2 | 1.70 | -11.4 |
| Mercury | ug/L | 62.7 | 60.3 | 0.00 | 0.00 | 0.0 | 0.00 | 0.0 | 0.00 | 0.0 |
| Nickel | ug/L | 61.1 | 58.8 | 0.1 | 0.0 | 0.1 | 0.0 | 0.1 | 0.0 | 0.0 |
| Selenium | ug/L | 62.5 | 61.4 | 1.2 | 0.7 | 0.2 | 0.5 | 0.2 | 0.1 | 0.0 |
| Silver | ug/L | 60.7 | 59.0 | 0.0 | 0.0 | 0.0 | 0.0 | 0.0 | 0.0 | 0.0 |
| Thallium | ug/L | 60.6 | 57.7 | 0.0 | 0.0 | 0.0 | 0.0 | 0.0 | 0.0 | 0.0 |
| Vanadium | ug/L | 22.7 | 21.0 | 0.3 | 0.1 | 0.1 | 0.1 | 0.1 | 0.00 | 0.0 |
| Zinc | ug/L | 60.0 | 57.6 | 0.8 | 1.0 | -0.6 | 0.2 | 1.2 | 0.80 | -1.8 |
| **Major ions, pH, TDS, and Hardness** | |  |  |  |  |  |  |  |  |  |
| Alkalinity | mg/L | 58.1 | 57.4 | 28.3 | 23.3 | 7.1 | 14.9 | 17.0 | 8.40 | -9.9 |
| Calcium | mg/L | 58.4 | 57.6 | 30.80 | 25.90 | 14.1 | 17.40 | 19.3 | 8.50 | -5.2 |
| Chloride | mg/L | 60.9 | 58.7 | 33.70 | 25.10 | 74.7 | 16.40 | 83.0 | 8.60 | -8.3 |
| Magnesium | mg/L | 58.1 | 57.0 | 26.20 | 21.80 | 4.0 | 15.80 | 5.2 | 5.90 | -1.2 |
| Potassium | mg/L | 58.1 | 49.1 | 1.6 | 1.3 | 1.7 | 0.9 | 1.7 | 0.40 | 0.0 |
| Sodium | mg/L | 57.9 | 57.0 | 26.4 | 20.1 | 44.8 | 14.0 | 49.0 | 6.10 | -4.1 |
| Sulfate | mg/L | 60.5 | 59.2 | 31.4 | 24.4 | 18.9 | 15.8 | 31.5 | 8.50 | -12.6 |
| pH, Lab | unitless | 59.1 | 59.4 | 20.3 | 17.5 | 0.0 | 10.5 | 0.2 | 7.00 | -0.1 |
| TDS | mg/L | 61.5 | 59.1 | 31.6 | 23.8 | 1.8 | 17.6 | 2.1 | 6.10 | -36.6 |
| Hardness | mg/L | 57.8 | 57.5 | 31.60 | 26.00 | 35.2 | 18.60 | 52.3 | 7.50 | -17.1 |

1. Recent (since 2000) water-quality trends in wells in the State Of California.

| Recent Trends (RT) | | | | Significant Trends | | | Positive Trends | | Negative Trends | |
| --- | --- | --- | --- | --- | --- | --- | --- | --- | --- | --- |
| Constituent | Units | Percent of wells tested | Percent of gridded area tested | Percent of tested wells with a trend | Percent of gridded area with a trend | Avg. Sen slope of tested area units/yr x 100 | Percent of gridded area with a trend | Avg. Sen slope of tested area units/yr x 100 | Percent of gridded area with a trend | Avg. Sen slope of tested area units/yr x 100 |
| **Nutrients** |  |  |  |  |  |  |  |  |  |  |
| Nitrate | mg/L as N | 69.9 | 76.3 | 47.3 | 36.2 | 2.8 | 22.3 | 5.2 | 13.9 | -2.4 |
| Nitrite | mg/L as N | 56.6 | 67.1 | 0.0 | 0.0 | -0.2 | 0.0 | 0.0 | 0.0 | -0.2 |
| **Radioactive constituents** | |  |  |  |  |  |  |  |  |  |
| Gross alpha | pCi/L | 43.9 | 54.6 | 11.0 | 10.0 | 0.0 | 4.9 | 2.1 | 5.1 | -2.1 |
| Gross beta | mg/L | 4.8 | 1.6 | 24.4 | 16.9 | -0.5 | 3.6 | 0.1 | 13.4 | -0.5 |
| Radium 226 | pCi/L | 8.7 | 5.7 | 0.9 | 3.1 | 0.0 | 0.00 | 0.0 | 3.1 | 0.0 |
| Radium 228 | pCi/L | 12.8 | 16.4 | 1.3 | 2.3 | 0.0 | 0.2 | 0.0 | 2.1 | 0.0 |
| Radium 226 + 228 | pCi/L | 2.2 | 1.3 | 0.0 | 0.0 | 0.0 | 0.0 | 0.0 | 0.00 | 0.0 |
| Uranium | pCi/L | 26.2 | 25.8 | 22.8 | 22.4 | -0.7 | 8.5 | 1.9 | 14.00 | -2.5 |
| **Trace elements** |  |  |  |  |  |  |  |  |  |  |
| Aluminum | ug/L | 49.5 | 53.2 | 0.70 | 0.60 | 0.3 | 0.40 | 5.5 | 0.10 | -5.2 |
| Antimony | ug/L | 50.8 | 53.3 | 0.00 | 0.00 | 0.0 | 0.00 | 0.0 | 0.00 | 0.0 |
| Arsenic | ug/L | 54.6 | 58.6 | 13.40 | 13.60 | -0.8 | 4.40 | 1.9 | 9.20 | -2.8 |
| Barium | ug/L | 47.0 | 52.0 | 5.30 | 3.90 | 7.9 | 2.70 | 13.2 | 1.20 | -5.3 |
| Beryllium | ug/L | 51.0 | 53.1 | 0.00 | 0.00 | 0.0 | 0.00 | 0.0 | 0.00 | 0.0 |
| Boron | ug/L | 23.1 | 21.6 | 13.30 | 9.90 | 30.0 | 6.40 | 45.1 | 3.40 | -15.2 |
| Cadmium | ug/L | 47.2 | 51.4 | 0.00 | 0.00 | 0.0 | 0.00 | 0.0 | 0.00 | 0.0 |
| Chromium (total) | ug/L | 46.8 | 52.8 | 2.10 | 2.10 | -0.4 | 0.80 | 0.4 | 1.20 | -0.8 |
| Copper | ug/L | 45.9 | 51.0 | 0.10 | 0.00 | -1.0 | 0.00 | 0.0 | 0.00 | -1.0 |
| Fluoride | mg/L | 44.7 | 52.6 | 16.00 | 11.70 | 0.0 | 5.30 | 0.1 | 6.40 | -0.1 |
| Iron | ug/L | 35.8 | 47.2 | 9.20 | 11.40 | -49.2 | 5.80 | 1.9 | 5.60 | -2.4 |
| Lead | ug/L | 42.6 | 45.0 | 0.00 | 0.00 | 0.0 | 0.00 | 0.0 | 0.00 | 0.0 |
| Manganese | ug/L | 45.2 | 53.2 | 7.00 | 7.20 | 14.3 | 4.00 | 39.2 | 3.20 | -24.9 |
| Mercury | ug/L | 46.3 | 50.0 | 0.00 | 0.00 | 0.0 | 0.00 | 0.0 | 0.00 | 0.0 |
| Nickel | ug/L | 51.0 | 53.0 | 0.2 | 0.1 | 0.2 | 0.0 | 0.3 | 0.0 | -0.1 |
| Selenium | ug/L | 46.7 | 51.7 | 1.4 | 1.0 | -0.1 | 0.5 | 0.1 | 0.6 | -0.3 |
| Silver | ug/L | 45.6 | 50.6 | 0.0 | 0.0 | 0.0 | 0.0 | 0.0 | 0.0 | 0.0 |
| Thallium | ug/L | 50.8 | 53.1 | 0.0 | 0.0 | 0.0 | 0.0 | 0.0 | 0.0 | 0.0 |
| Vanadium | ug/L | 22.3 | 20.5 | 0.5 | 0.5 | 0.2 | 0.2 | 0.3 | 0.30 | -0.1 |
| Zinc | ug/L | 45.0 | 50.0 | 0.6 | 0.8 | -3.6 | 0.3 | 2.0 | 0.50 | -5.6 |
| **Major ions, pH, TDS, and Hardness** | |  |  |  |  |  |  |  |  |  |
| Alkalinity | mg/L | 43.6 | 48.6 | 23.1 | 19.9 | 1.3 | 10.5 | 16.5 | 9.40 | -15.2 |
| Calcium | mg/L | 43.8 | 50.3 | 29.50 | 24.80 | 3.7 | 14.20 | 12.5 | 10.60 | -8.7 |
| Chloride | mg/L | 45.0 | 49.7 | 33.90 | 27.80 | 98.9 | 17.80 | 1.1 | 10.10 | -10.3 |
| Magnesium | mg/L | 43.6 | 49.2 | 25.00 | 21.40 | 5.5 | 13.10 | 8.1 | 8.40 | -2.5 |
| Potassium | mg/L | 43.7 | 41.1 | 1.6 | 1.5 | 1.4 | 0.9 | 1.4 | 0.60 | 0.0 |
| Sodium | mg/L | 43.3 | 49.1 | 24.7 | 19.0 | 42.8 | 11.6 | 47.4 | 7.40 | -4.6 |
| Sulfate | mg/L | 44.8 | 50.5 | 32.4 | 25.1 | 8.6 | 15.8 | 30.5 | 9.30 | -21.9 |
| pH, Lab | unitless | 43.4 | 51.4 | 24.7 | 19.6 | 0.2 | 14.3 | 0.3 | 5.30 | -0.1 |
| TDS | mg/L | 46.1 | 51.2 | 29.6 | 23.2 | 1.8 | 14.2 | 2.3 | 8.90 | -51.0 |
| Hardness | mg/L | 43.3 | 49.0 | 29.00 | 26.40 | 38.6 | 16.00 | 66.5 | 10.30 | -27.9 |

1. Reversal of water-quality trends in wells in the State Of California (all available data: 1970 – 2014).

| Trend Reversals (TRV) | | | | Significant Trends | | | Positive Trends | | Negative Trends | |
| --- | --- | --- | --- | --- | --- | --- | --- | --- | --- | --- |
| Constituent | Units | Percent of wells tested | Percent of gridded area tested | Percent of tested wells with a trend | Percent of gridded area with a trend | Avg. Sen slope of tested area units/yr x 100 | Percent of gridded area with a trend | Avg. Sen slope of tested area units/yr x 100 | Percent of gridded area with a trend | Avg. Sen slope of tested area units/yr x 100 |
| **Nutrients** |  |  |  |  |  |  |  |  |  |  |
| Nitrate | mg/L as N | 68.0 | 73.1 | 8.1 | 5.1 | -0.3 | 1.8 | 0.4 | 3.2 | -0.7 |
| Nitrite | mg/L as N | 25.8 | 42.3 | 0.0 | 0.0 | 0.0 | 0.0 | 0.0 | 0.0 | 0.0 |
| **Radioactive constituents** | |  |  |  |  |  |  |  |  |  |
| Gross alpha | pCi/L | 44.4 | 45.2 | 2.4 | 2.3 | -0.4 | 0.7 | 0.1 | 1.7 | -0.5 |
| Gross beta | mg/L | 7.5 | 2.3 | 3.4 | 1.5 | 0.0 | 1.0 | 0.0 | 0.5 | 0.0 |
| Radium 226 | pCi/L | 4.4 | 3.3 | 0.0 | 0.0 | 0.0 | 0.00 | 0.0 | 0.0 | 0.0 |
| Radium 228 | pCi/L | 2.1 | 4.4 | 0.0 | 0.0 | 0.0 | 0.0 | 0.0 | 0.0 | 0.0 |
| Radium 226 + 228 | pCi/L | 1.0 | 1.1 | 0.0 | 0.0 | 0.0 | 0.0 | 0.0 | 0.00 | 0.0 |
| Uranium | pCi/L | 16.2 | 17.6 | 5.5 | 2.4 | -0.3 | 0.8 | 0.2 | 1.70 | -0.5 |
| **Trace elements** |  |  |  |  |  |  |  |  |  |  |
| Aluminum | ug/L | 26.4 | 31.7 | 0.10 | 0.00 | 0.8 | 0.00 | 0.8 | 0.00 | 0.0 |
| Antimony | ug/L | 18.6 | 23.5 | 0.00 | 0.00 | 0.0 | 0.00 | 0.0 | 0.00 | 0.0 |
| Arsenic | ug/L | 31.4 | 41.1 | 2.20 | 2.20 | -0.2 | 1.20 | 0.2 | 1.10 | -0.3 |
| Barium | ug/L | 31.3 | 34.0 | 0.50 | 0.30 | -1.1 | 0.10 | 0.2 | 0.20 | -1.3 |
| Beryllium | ug/L | 18.4 | 23.1 | 0.00 | 0.00 | 0.0 | 0.00 | 0.0 | 0.00 | 0.0 |
| Boron | ug/L | 13.1 | 10.1 | 1.20 | 1.50 | 0.1 | 1.00 | 1.2 | 0.50 | -1.1 |
| Cadmium | ug/L | 30.2 | 31.9 | 0.00 | 0.00 | 0.0 | 0.00 | 0.0 | 0.00 | 0.0 |
| Chromium (total) | ug/L | 32.6 | 34.9 | 0.30 | 0.20 | -0.4 | 0.00 | 0.0 | 0.20 | -0.4 |
| Copper | ug/L | 33.5 | 34.8 | 0.00 | 0.00 | 0.0 | 0.00 | 0.0 | 0.00 | 0.0 |
| Fluoride | mg/L | 32.6 | 35.9 | 3.90 | 1.80 | 0.0 | 1.00 | 0.0 | 0.80 | 0.0 |
| Iron | ug/L | 24.0 | 33.0 | 2.10 | 3.10 | -15.1 | 2.40 | 11.6 | 0.70 | -26.7 |
| Lead | ug/L | 27.9 | 29.5 | 0.00 | 0.00 | 0.0 | 0.00 | 0.0 | 0.00 | 0.0 |
| Manganese | ug/L | 35.3 | 40.9 | 1.30 | 0.80 | -3.6 | 0.30 | 0.8 | 0.50 | -4.3 |
| Mercury | ug/L | 31.5 | 33.6 | 0.00 | 0.00 | 0.0 | 0.00 | 0.0 | 0.00 | 0.0 |
| Nickel | ug/L | 18.6 | 23.3 | 0.0 | 0.0 | 0.0 | 0.0 | 0.0 | 0.0 | 0.0 |
| Selenium | ug/L | 31.5 | 33.3 | 0.3 | 0.4 | -0.1 | 0.0 | 0.0 | 0.4 | -0.1 |
| Silver | ug/L | 32.0 | 33.4 | 0.0 | 0.0 | 0.0 | 0.0 | 0.0 | 0.0 | 0.0 |
| Thallium | ug/L | 18.3 | 22.5 | 0.0 | 0.0 | 0.0 | 0.0 | 0.0 | 0.0 | 0.0 |
| Vanadium | ug/L | 5.5 | 4.2 | 0.0 | 0.0 | 0.0 | 0.0 | 0.0 | 0.00 | 0.0 |
| Zinc | ug/L | 32.9 | 34.3 | 0.0 | 0.1 | -4.7 | 0.0 | 0.0 | 0.10 | -4.7 |
| **Major ions, pH, TDS, and Hardness** | |  |  |  |  |  |  |  |  |  |
| Alkalinity | mg/L | 32.6 | 35.2 | 4.8 | 2.9 | 0.2 | 1.5 | 2.1 | 1.40 | -1.9 |
| Calcium | mg/L | 33.4 | 36.6 | 5.30 | 3.30 | 0.1 | 1.60 | 1.4 | 1.70 | -1.3 |
| Chloride | mg/L | 34.0 | 35.7 | 5.70 | 3.80 | -0.9 | 1.60 | 1.3 | 2.20 | -2.2 |
| Magnesium | mg/L | 32.8 | 35.5 | 4.00 | 2.90 | -0.1 | 1.00 | 0.3 | 1.90 | -0.4 |
| Potassium | mg/L | 32.1 | 29.6 | 0.2 | 0.5 | 0.1 | 0.5 | 0.1 | 0.00 | 0.0 |
| Sodium | mg/L | 32.5 | 34.6 | 3.9 | 3.5 | 0.7 | 1.9 | 1.5 | 1.60 | -0.8 |
| Sulfate | mg/L | 33.5 | 34.9 | 5.0 | 3.6 | -0.5 | 1.7 | 2.3 | 2.00 | -2.7 |
| pH, Lab | unitless | 33.2 | 36.3 | 6.2 | 3.8 | 0.0 | 3.0 | 0.0 | 0.80 | 0.0 |
| TDS | mg/L | 35.0 | 35.9 | 5.5 | 3.5 | -2.7 | 1.5 | 7.4 | 2.00 | -10.1 |
| Hardness | mg/L | 32.7 | 35.2 | 5.30 | 3.80 | -0.4 | 1.40 | 3.8 | 2.40 | -4.3 |

1. Seasonal water-quality trends in wells in the State Of California (all available data: 1970 – 2014).

| Seasonal Trends (ST) | | | | Significant Trends | | | Positive Trends | | Negative Trends | |
| --- | --- | --- | --- | --- | --- | --- | --- | --- | --- | --- |
| Constituent | Units | Percent of wells tested | Percent of gridded area tested | Percent of tested wells with a trend | Percent of gridded area with a trend | Avg. Sen slope of tested area units/yr x 100 | Percent of gridded area with a trend | Avg. Sen slope of tested area units/yr x 100 | Percent of gridded area with a trend | Avg. Sen slope of tested area units/yr x 100 |
| **Nutrients** |  |  |  |  |  |  |  |  |  |  |
| Nitrate | mg/L as N | 47.2 | 63.9 | 7.4 | 4.4 | 0.2 | 3.2 | 0.3 | 1.2 | -0.2 |
| Nitrite | mg/L as N | 11.9 | 19.7 | 0.0 | 0.0 | 0.0 | 0.0 | 0.0 | 0.0 | 0.0 |
| **Radioactive constituents** | |  |  |  |  |  |  |  |  |  |
| Gross alpha | pCi/L | 37.2 | 40.3 | 0.6 | 0.7 | 0.0 | 0.6 | 0.1 | 0.1 | 0.0 |
| Gross beta | mg/L | 5.2 | 1.9 | 2.0 | 3.1 | -0.1 | 0.0 | 0.0 | 3.1 | -0.1 |
| Radium 226 | pCi/L | 2.2 | 2.4 | 0.0 | 0.0 | 0.0 | 0.00 | 0.0 | 0.0 | 0.0 |
| Radium 228 | pCi/L | 1.2 | 5.0 | 0.0 | 0.0 | 0.0 | 0.0 | 0.0 | 0.0 | 0.0 |
| Radium 226 + 228 | pCi/L | 0.8 | 1.2 | 0.0 | 0.0 | 0.0 | 0.0 | 0.0 | 0.00 | 0.0 |
| Uranium | pCi/L | 12.4 | 16.4 | 2.3 | 1.2 | 0.0 | 1.1 | 0.0 | 0.10 | 0.0 |
| **Trace elements** |  |  |  |  |  |  |  |  |  |  |
| Aluminum | ug/L | 8.2 | 14.8 | 0.10 | 0.10 | 0.0 | 0.10 | 0.0 | 0.00 | 0.0 |
| Antimony | ug/L | 6.0 | 10.5 | 0.00 | 0.00 | 0.0 | 0.00 | 0.0 | 0.00 | 0.0 |
| Arsenic | ug/L | 15.0 | 28.5 | 2.50 | 2.70 | 0.2 | 1.10 | 0.3 | 1.50 | -0.1 |
| Barium | ug/L | 11.4 | 18.8 | 0.80 | 0.70 | 0.0 | 0.40 | 0.0 | 0.30 | -0.1 |
| Beryllium | ug/L | 5.8 | 10.0 | 0.00 | 0.00 | 0.0 | 0.00 | 0.0 | 0.00 | 0.0 |
| Boron | ug/L | 6.0 | 5.4 | 2.60 | 2.10 | 0.2 | 1.60 | 1.0 | 0.50 | -0.8 |
| Cadmium | ug/L | 10.6 | 17.1 | 0.00 | 0.00 | 0.0 | 0.00 | 0.0 | 0.00 | 0.0 |
| Chromium (total) | ug/L | 13.9 | 21.1 | 0.20 | 0.30 | 0.0 | 0.30 | 0.0 | 0.00 | 0.0 |
| Copper | ug/L | 14.1 | 20.5 | 0.00 | 0.00 | 0.0 | 0.00 | 0.0 | 0.00 | 0.0 |
| Fluoride | mg/L | 14.4 | 22.5 | 2.00 | 1.10 | 0.0 | 0.40 | 0.0 | 0.80 | 0.0 |
| Iron | ug/L | 11.3 | 22.2 | 1.20 | 1.20 | -0.3 | 1.00 | 2.2 | 0.10 | -2.4 |
| Lead | ug/L | 9.7 | 15.5 | 0.00 | 0.00 | 0.0 | 0.00 | 0.0 | 0.00 | 0.0 |
| Manganese | ug/L | 17.3 | 29.1 | 0.80 | 0.80 | 0.3 | 0.50 | 0.5 | 0.30 | -0.1 |
| Mercury | ug/L | 11.7 | 17.9 | 0.00 | 0.00 | 0.0 | 0.00 | 0.0 | 0.00 | 0.0 |
| Nickel | ug/L | 5.8 | 10.0 | 0.0 | 0.0 | 0.0 | 0.0 | 0.0 | 0.0 | 0.0 |
| Selenium | ug/L | 11.8 | 18.2 | 0.3 | 0.1 | 0.0 | 0.1 | 0.0 | 0.0 | 0.0 |
| Silver | ug/L | 12.1 | 18.5 | 0.0 | 0.0 | 0.0 | 0.0 | 0.0 | 0.0 | 0.0 |
| Thallium | ug/L | 5.8 | 9.8 | 0.0 | 0.0 | 0.0 | 0.0 | 0.0 | 0.0 | 0.0 |
| Vanadium | ug/L | 2.7 | 2.6 | 0.0 | 0.0 | 0.0 | 0.0 | 0.0 | 0.00 | 0.0 |
| Zinc | ug/L | 13.4 | 20.0 | 0.1 | 0.0 | -0.1 | 0.0 | 0.0 | 0.00 | -0.1 |
| **Major ions, pH, TDS, and Hardness** | | |  |  |  |  |  |  |  |  |
| Alkalinity | mg/L | 14.2 | 21.1 | 4.3 | 3.5 | 0.5 | 2.6 | 0.8 | 0.90 | -0.3 |
| Calcium | mg/L | 15.2 | 22.9 | 6.60 | 5.60 | 0.6 | 3.80 | 0.9 | 1.80 | -0.3 |
| Chloride | mg/L | 14.5 | 22.0 | 6.60 | 6.10 | 1.1 | 3.00 | 1.3 | 3.10 | -0.2 |
| Magnesium | mg/L | 14.0 | 21.7 | 4.50 | 3.90 | 0.2 | 2.90 | 0.2 | 1.00 | 0.0 |
| Potassium | mg/L | 13.1 | 17.1 | 0.1 | 0.2 | 0.0 | 0.2 | 0.0 | 0.00 | 0.0 |
| Sodium | mg/L | 13.7 | 21.4 | 3.9 | 5.6 | 0.3 | 4.6 | 0.4 | 1.00 | -0.1 |
| Sulfate | mg/L | 14.4 | 21.9 | 5.8 | 3.6 | 0.6 | 3.0 | 1.4 | 0.60 | -0.8 |
| pH, Lab | unitless | 15.0 | 23.6 | 3.2 | 1.7 | 0.0 | 0.9 | 0.0 | 0.80 | 0.0 |
| TDS | mg/L | 16.0 | 23.6 | 6.0 | 5.0 | 1.0 | 4.2 | 6.0 | 0.80 | -5.1 |
| Hardness | mg/L | 14.1 | 21.1 | 5.70 | 5.30 | 2.2 | 4.00 | 2.9 | 1.30 | -0.7 |

1. Proportions of physiographic provinces with improving and degrading groundwater quality conditions. Numbers are percentages of the total area covered by the grid cells in each province.

| Physiographic Province | Constituent | Improving | | | Indeter minate | Degrading | | | Area of Grid Tested |
| --- | --- | --- | --- | --- | --- | --- | --- | --- | --- |
|  |  | Hi | Moderate | Low |  | Low | Moderate | Hi |  |
| Desert - Basin and Range (DBR) | Arsenic | 1.8 | 2.1 | 2.5 | 0.1 | 1.5 | 2.7 | 2.5 | 68.1 |
| Klamath Mountains - Cascade Range and Modoc Plateau (KCM) | Arsenic | 0.6 | 0.3 | 4.2 |  |  | 0.9 |  | 28.5 |
| Northern Coast Ranges | Arsenic | 3.3 | 5.4 | 1.7 |  | 1.2 | 0.6 |  | 71.9 |
| Sacramento Valley (SAC) | Arsenic | 4.9 | 2.9 | 2.3 | 0.3 | 0.4 | 3.1 | 1.8 | 75.6 |
| San Diego (SND) | Arsenic |  | 2.8 | 1.5 |  | 2.3 |  |  | 58.0 |
| San Joaquin Valley (SJV) | Arsenic | 3.8 | 5.0 | 6.6 | 0.6 | 1.7 | 1.4 | 2.2 | 79.5 |
| Sierra Nevada (SNR) | Arsenic | 0.5 | 5.7 | 0.5 | 0.1 | 2.0 | 0.1 |  | 82.5 |
| Southern Coast Ranges (SCR) | Arsenic | 0.8 | 1.3 | 3.0 | 0.1 | 1.4 | 1.0 | 0.6 | 74.2 |
| Transverse and Selected Peninsular Range (TSPR) | Arsenic | 0.3 | 3.0 | 2.5 | 0.1 | 1.7 | 0.2 |  | 83.9 |
| Desert - Basin and Range (DBR) | Gross alpha |  | 3.0 | 1.4 | 0.3 | 7.3 | 1.7 |  | 72.2 |
| Klamath Mountains - Cascade Range and Modoc Plateau (KCM) | Gross alpha |  |  |  |  |  | 0.5 |  | 39.1 |
| Northern Coast Ranges | Gross alpha |  |  |  |  | 1.2 |  |  | 56.3 |
| Sacramento Valley (SAC) | Gross alpha |  |  | 0.1 |  | 2.3 |  |  | 68.7 |
| San Diego (SND) | Gross alpha |  | 4.9 | 7.5 | 0.7 | 4.0 | 4.4 |  | 62.2 |
| San Joaquin Valley (SJV) | Gross alpha | 0.4 | 1.5 | 4.1 | 0.4 | 2.4 | 1.1 | 1.8 | 73.5 |
| Sierra Nevada (SNR) | Gross alpha |  | 6.8 | 1.3 |  | 1.0 | 0.9 |  | 78.4 |
| Southern Coast Ranges (SCR) | Gross alpha |  | 0.9 | 2.3 | 0.4 | 3.8 | 0.2 |  | 70.1 |
| Transverse and Selected Peninsular Range (TSPR) | Gross alpha | 0.3 | 2.4 | 4.5 | 1.0 | 4.0 | 0.7 | 0.3 | 83.5 |
| Desert - Basin and Range (DBR) | TDS |  | 3.0 | 5.2 | 0.2 | 12.0 | 1.3 | 1.2 | 61.6 |
| Klamath Mountains - Cascade Range and Modoc Plateau (KCM) | TDS |  |  | 11.1 |  | 13.2 |  |  | 13.4 |
| Northern Coast Ranges | TDS |  | 0.1 | 4.5 | 2.5 | 7.1 | 1.2 |  | 59.3 |
| Sacramento Valley (SAC) | TDS |  | 2.5 | 2.0 | 1.0 | 6.0 | 1.0 |  | 51.6 |
| San Diego (SND) | TDS | 0.4 | 5.3 | 6.3 | 3.1 | 7.1 | 5.3 | 3.6 | 61.8 |
| San Joaquin Valley (SJV) | TDS |  | 1.7 | 5.3 | 0.9 | 13.8 | 2.1 | 0.8 | 69.4 |
| Sierra Nevada (SNR) | TDS |  | 0.1 | 7.4 | 1.2 | 7.8 |  |  | 77.8 |
| Southern Coast Ranges (SCR) | TDS | 1.0 | 5.5 | 3.7 | 1.4 | 9.6 | 3.6 | 1.2 | 70.3 |
| Transverse and Selected Peninsular Range (TSPR) | TDS | 0.7 | 5.3 | 3.3 | 2.4 | 18.9 | 7.8 | 2.5 | 83.2 |
| Desert - Basin and Range (DBR) | Nitrate |  | 1.3 | 12.3 | 2.1 | 24.3 | 2.7 |  | 80.5 |
| Klamath Mountains - Cascade Range and Modoc Plateau (KCM) | Nitrate |  | 0.3 | 3.1 | 0.5 | 5.4 | 0.5 |  | 92.2 |
| Northern Coast Ranges | Nitrate |  | 1.2 | 11.7 | 2.6 | 6.2 | 1.0 |  | 95.3 |
| Sacramento Valley (SAC) | Nitrate |  | 4.9 | 9.3 | 2.1 | 24.5 | 3.1 | 0.6 | 98.5 |
| San Diego (SND) | Nitrate |  | 1.6 | 4.5 | 1.6 | 15.4 | 0.9 | 0.6 | 86.5 |
| San Joaquin Valley (SJV) | Nitrate |  | 5.1 | 5.6 | 1.2 | 23.5 | 8.8 | 2.0 | 92.3 |
| Sierra Nevada (SNR) | Nitrate |  | 1.0 | 5.3 | 1.3 | 8.0 | 0.8 |  |  |
| Southern Coast Ranges (SCR) | Nitrate |  | 4.9 | 13.0 | 2.9 | 17.4 | 4.5 | 1.4 | 91.2 |
| Transverse and Selected Peninsular Range (TSPR) | Nitrate | 1.4 | 8.7 | 13.0 | 2.1 | 19.3 | 7.0 | 3.3 | 91.8 |
